# Synthesis, Biological Evaluation, and Molecular Docking Studies of Novel Isatin-Thiazole Derivatives as α-Glucosidase Inhibitors

**DOI:** 10.3390/molecules22040659

**Published:** 2017-04-20

**Authors:** Zhenzhen Xie, Guangcheng Wang, Jing Wang, Ming Chen, Yaping Peng, Luyao Li, Bing Deng, Shan Chen, Wenbiao Li

**Affiliations:** College of Chemistry and Chemical Engineering, Hunan Engineering Laboratory for Analyse and Drugs Development of Ethnomedicine in Wuling Mountains, Jishou University, Jishou 416000, China; xiezz1993@126.com (Z.X.); wangjingjsu@sina.com (J.W.); mingchen940517@163.com (M.C.); yapingpeng17@163.com (Y.P.); liluyao15@yeah.net (L.L.); dengbingfish@163.com (B.D.); chenshan20142014@163.com (S.C.); liwenbiao0926@yeah.net (W.L.)

**Keywords:** α-glucosidase inhibitor, molecular docking, isatin, thiazole, diabetic

## Abstract

A series of novel isatin-thiazole derivatives were synthesized and screened for their in vitro α-glucosidase inhibitory activity. These compounds displayed a varying degree of α-glucosidase inhibitory activity with IC_50_ ranging from 5.36 ± 0.13 to 35.76 ± 0.31 μm as compared to the standard drug acarbose (IC_50_ = 817.38 ± 6.27 μm). Among the series, compound **6p** bearing a hydroxyl group at the 4-position of the right phenyl and 2-fluorobenzyl substituent at the *N*1-positions of the 5-methylisatin displayed the highest inhibitory activity with an IC_50_ value of 5.36 ± 0.13 μm. Molecular docking studies revealed the existence of hydrophobic interaction, CH-π interaction, arene-anion interaction, arene-cation interaction, and hydrogen bond between these compounds and α-glucosidase enzyme.

## 1. Introduction

Diabetes mellitus is a group of metabolic disorders of carbohydrate metabolism characterized by high blood glucose levels (hyperglycemia), resulting from defects in insulin secretion, insulin action, or both [1]. Currently, there are an estimated 422 million people have diabetes mellitus in the world, according to the latest 2016 data from the World Health Organization (WHO) [2]. In diabetic patients, untreated and chronic hyperglycemia can cause serious complications, such as heart disease, stroke, blindness, high blood pressure, kidney disease, and nervous system disease [3]. α-Glucosidase is a membrane-bound enzyme at the epithelium of the small intestine and hydrolyzes terminal non-reducing 1–4 linked α-glucose residues to release monomeric glucose molecules which is mainly responsible to cause hyperglycemia [4]. Inhibition of α-glucosidase activity can delay carbohydrate absorption and have been used as one of the therapeutic approaches for the treatment of diabetes [4,5]. Some α-glucosidase inhibitors (acarbose, miglitol, and voglibose) have been approved for clinical use and also used as anticancer [6], anti-HIV [7], and anti-hepatitis agents [8]. Therefore, design and synthesis of small molecules as α-glucosidase inhibitors is an important research area in medicinal chemistry.

**Figure 1:**
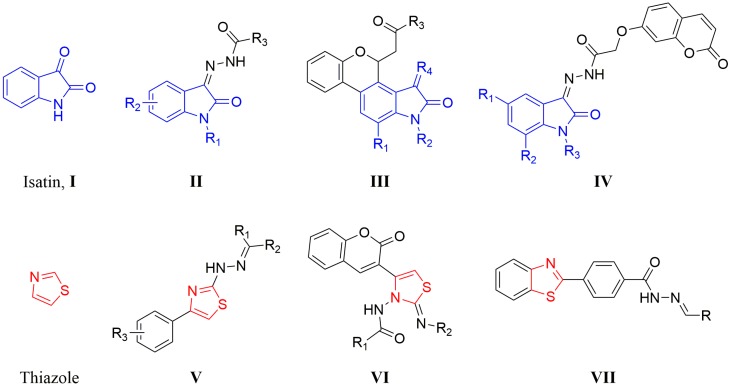
The chemical structures of the reported α-glucosidase inhibitors containing isatin or thiazole moiety.

Isatin (1*H*-indole-2,3-dione, **I**) is the reference compound of an important class of nitrogen-containing aromatic heterocyclic compounds, which have been found in many plants and human blood and tissue [9]. Isatin has emerged as a promising nucleus and attracted increasing attention in medicinal chemistry and drug discovery over the past decade [10]. Previous literature reports indicated that isatin and its derivatives have diverse types of biological activity, including anticancer [11], antibacterial [12], antiviral [13], anticonvulsant [14], anti-inflammatory [15], and antifungal activity [16]. Notably, some isatin derivatives have been approved for clinic use such as sunitinib, toceranib, and nintedanib. Furthermore, Rahim et al. reported the synthesis of isatin based Schiff bases **II** (Figure 1) which showed excellent inhibitory potential many fold better than the standard acarbose [17]. Sun et al. reported the synthesis of tetracyclic oxindole derivatives **III** (Figure 1) and the most active compound (IC_50_ = 0.7 μm) was about 170 times as active as acarbose [18]. Recently, we have also synthesized a series of coumarin-isatin derivatives **IV** (Figure 1), and some of the obtained compound exhibited excellent α-glucosidase inhibition activity [19].

On the other hand, thiazole derivatives are considered as another important class of heterocyclic compounds, which displayed a wide range of pharmacological activities such as anti-inflammatory [20], anticancer [21], anticonvulsant [22], and antibacterial [23]. It is interesting that numerous studies pointed out thiazole could be used as a useful moiety in the design of potent α-glucosidase inhibitors [24,25,26,27,28]. For example, compound series **V** [25], **VI** [28], and **VII** [26] displayed potent α-glucosidase inhibitory activity (Figure 1).

Over the years, molecular hybrid-based approaches had been exploited by researchers to discover some promising chemical architectures which containing two or more bioactive pharmacophores [29,30]. Using this approach, and as part of our ongoing effort to develop potent α-glucosidase inhibitors [31,32,33,34,35], herein we report the design and synthesis of a series of novel isatin-thiazole derivatives containing isatin and thiazole moieties. The synthesized compounds were evaluated for their inhibitory activity against α-glucosidase. Furthermore, molecular docking was also performed to investigate the interaction of inhibitors with enzymes.

## 2. Results and Discussion

### 2.1. Chemistry

**Scheme 1:**
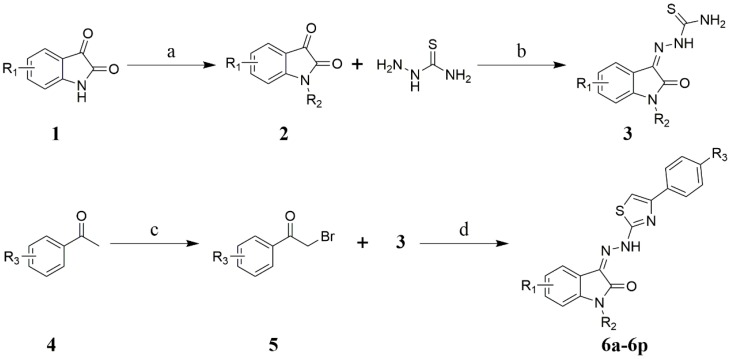
*Reagents and conditions*: (**a**) R_2_X, K_2_CO_3_, DMF, room temperature, 2 h; (**b**) EtOH, 45 °C, 3 h; (**c**) *p*-MeC_6_H_4_SO_3_H, NBS, CH_3_CN, reflux, 2 h; (**d**) EtOH, reflux, 2 h.

The synthesis of isatin-thiazole derivatives **6a**–**6p** was shown in Scheme 1. Reaction of commercially available isatins **1** with various alkyl halides in the presence of K_2_CO_3_ in DMF provided *N*-alkyl isatins **2**. Isatins (**1** and **2**) were stirred with thiosemicarbazide in ethanol at 45 °C for 3 h to provide the isatin thiosemicarbazones **3**. Various substitutions of acetophenone **4** were treated with NBS in the presence of *p*-toluenesulfonic acid in acetonitrile to give α-bromoacetophenone **5**. α-bromoacetophenone **5**, with appropriate isatin thiosemicarbazones **3**, was condensed in refluxing ethanol for 2 h to afford the corresponding isatin-thiazole derivatives **6a**–**6p** in moderate to high yield (52.4–78.4%). All of the title compounds **6a**–**6p** have not yet been reported in the literature.

The structures of all of the title compounds **6a**–**6p** were characterized by ^1^H-NMR spectra. The ^1^H-NMR spectrum of **6a** exhibited two singlet signals at 11.36 and 13.33 ppm, attributed to the protons of NH-indolin-2-one and =NNH group, respectively. Two doublet signals at 6.97 ppm (*J* = 8.4 Hz) and 7.52 ppm (*J* = 2.0 Hz) were attributed to C7-H and C4-H of isatin ring, respectively. The double-double peak of C6-H of the isatin ring was observed at 7.36 ppm with a coupling constant of 8.4 Hz and 2.0 Hz. Two double peaks at 7.61 and 7.85 ppm with coupling constant of 8.4 Hz were attributed to the aromatic protons of C3,5-H and C2,6-H, respectively. The hydrogen atom of thiazole ring appeared as a singlet signal at 7.74 ppm. The ^1^H-NMR spectrum of all new compounds consistent with their structures. Moreover, in their ^13^C-NMR spectra, the number of signals equals the number of different carbons in the molecule (Supplementary Materials).

### 2.2. α-Glucosidase Inhibition Assay

All the synthetic compounds **6a**–**6p** were screened for their in vitro α-glucosidase inhibitory activity (Table 1). The results were shown that all the tested compounds displayed potent to moderate α-glucosidase inhibitory activity with IC_50_ ranging from 5.36 ± 0.13 to 35.76 ± 0.31 μm more potent than the standard drug acarbose (IC_50_ = 817.38 ± 6.27 μm [17,36]). Among the series, compounds **6a**, **6g**, **6h**, **6i**, **6j**, **6l**, **6m**, **6n**, and **6p** displayed potent inhibitory activity with IC_50_ values of 6.87 ± 0.14, 5.98 ± 0.12, 6.12 ± 0.15, 7.51 ± 0.17, 6.51 ± 0.13, 8.33 ± 0.18, 7.17 ± 0.15, 6.36 ± 0.12, and 5.36 ± 0.13 μm. In particular, compound **6p** (IC_50_ = 5.36 ± 0.13 μm) with a hydroxyl group at the 4-position of the right phenyl ring and methyl and 2-fluorobenzyl groups at 5- and N1-positions of the isatin ring, was found to be the most active compound. In comparison to compound **6p**, a decrease in activity was observed for **6i** (IC_50_ = 7.51 ± 0.17) in which 2-fluorobenzyl groups is replaced with hydrogen at N1-positions of the isatin ring. Compounds **6c**, **6k**, and **6o** also displayed good inhibition with IC_50_ value 10.34 ± 0.17, 15.68 ± 0.24, and 11.78 ± 0.21 μm, respectively. Other compounds displayed low α-glucosidase inhibitory activity. The binding interactions of the most active compounds with α-glucosidase were confirmed through molecular docking studies.

### 2.3. Homology Model

The crystallographic structure of *Saccharomyces cerevisiae* α-glucosidase enzyme has not been reported. To understand the ligand-enzyme interactions, the 3D structure of α-glucosidase was built by means of modeller 9.15 homology modeling software (http://salilab.org/modeller/). The sequence in FASTA format of α-glucosidase was retrieved from UniProt (access code P53341). The crystallographic structure of *Saccharomyces cerevisiae* isomaltase (PDB ID: 3AJ7, Resolution 1.30 Å) with 72.4% of sequence identity with the target was selected as the template for homology modeling [37]. The quality of homology model was verified by PROCHECK (http://services.mbi.ucla.edu/PROCHECK/). The result was shown that the model could be used to study the interactions between this class of compounds and the active site of α-glucosidase [35].

### 2.4. Molecular Docking

**Figure 2:**
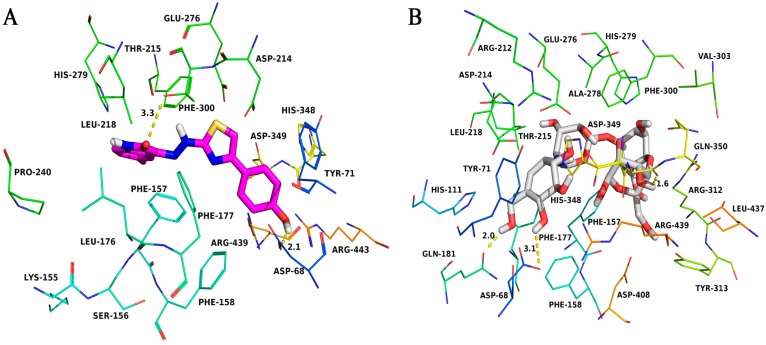
Compound **6i** (**A**) and acarbose (**B**) was docked to the binding pocket of the *Saccharomyces cerevisiae* α-glucosidase.

The theoretical binding mode between **6i** and *Saccharomyces cerevisiae* α-glucosidase was shown in Figure 2. Compound **6i** adopted a “V-shaped” conformation in the pocket of the α-glucosidase. The indolin-2-one scaffold of **6i** located at the hydrophobic pocket, surrounded by the residues Phe-157, Leu-176, Pro-240, Phe-300, and Leu-218, forming a stable hydrophobic binding. Detailed analysis showed that the indolin-2-one scaffold of **6i** formed CH-π interaction with the residue Phe-157. In addition, the 4-hydroxylphenyl group of **6i** formed CH-π interactions with the residues Phe-158 and Tyr-71, and arene-anion interactions with the residues Asp-68 and Asp-349. Also, the arene-cation interactions were observed between the 4-hydroxylphenyl group of **6i** and the residues Arg-439 and Arg-443. It was shown that the residues Thr-215 (bond length: 3.3 Å) and Asp-68 (length: 2.1 Å) formed two hydrogen bonds with **6i**, which was the main interaction between **6i** and α-glucosidase. On the other hand, molecular docking study of the standard drug acarbose with α-glucosidase was also performed (Figure 2B). The results were shown that compound **6i** (binding energy was about −9.2 kcal mol^−1^) has a similar binding affinity as compared to standard drug acarbose (binding energy was about −6.8 kcal mol^−1^).

**Figure 3:**
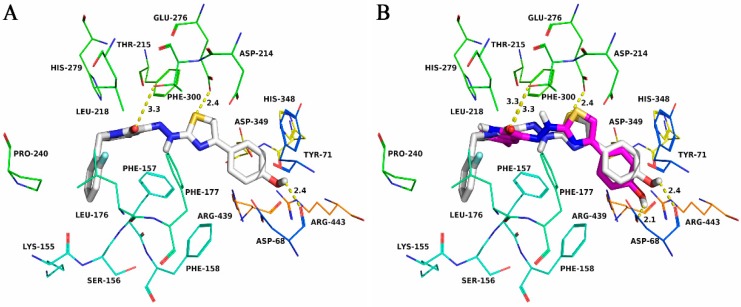
(**A**) Compound **6p** was docked to the binding pocket of the *Saccharomyces cerevisiae* α-glucosidase. (**B**) Compounds **6i** and **6p** were docked to the binding pocket of the *Saccharomyces cerevisiae* α-glucosidase (overlapped).

To explain the activity order of **6i** and **6p** against α-glucosidase in the molecular level, **6p** was further docked into the binding pocket of α-glucosidase, and the theoretical binding mode between **6p** and α-glucosidase was shown in Figure 3A. The interaction between **6p** and α-glucosidase was nearly the same as the compound **6i** (Figure 3B). The main difference was that the 2-fluorophenyl group of **6p** formed extra hydrophobic interactions with the residues Phe-157, Leu-176, Pro-240, and Leu-218, and formed two extra hydrogen bonds with the residue Glu-276 (length: 2.4 Å) and Asp-68 (length: 2.4 Å), which made **6p** was more active than **6i** against α-glucosidase (Figure 3B). In addition, the estimated binding energies were −9.2 kcal mol^−1^ for **6i** and −10.1 kcal mol^−1^ for **6p**, respectively, which was consistent with the results of the in vitro α-glucosidase inhibitory activity.

**Figure 4:**
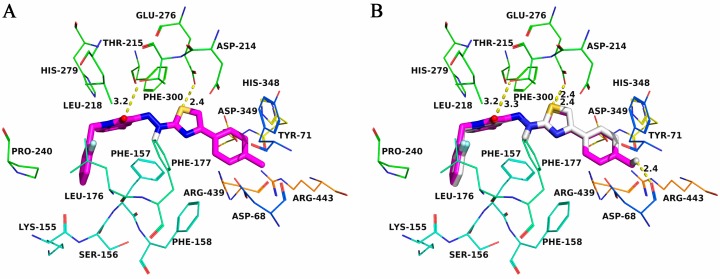
(**A**) Compound **6b** was docked to the binding pocket of the *Saccharomyces cerevisiae* α-glucosidase. (**B**) Compounds **6b** and **6p** were docked to the binding pocket of the *Saccharomyces cerevisiae* α-glucosidase (overlapped).

To explain the activity order of **6b** and **6p** against α-glucosidase in the molecular level, **6b** was further docked into the binding pocket of α-glucosidase, and the theoretical binding mode between **6b** and α-glucosidase was shown in Figure 4A. Compound **6b** adopted a ‘V-shaped’ conformation in the pocket of the α-glucosidase. The 2-fluorophenylindolin-2-one group of **6b** stretched into the hydrophobic pocket that consisted of Phe-157, Leu-176, Pro-240, Phe-300, and Leu-218, forming a stable hydrophobic binding. Detailed analysis showed that the indolin-2-one scaffold of **6b** formed CH-π interaction with the residue Phe-157. In addition, the 4-methylphenyl group of **6b** formed CH-π interactions with the residues Phe-158 and Tyr-71, and arene-anion interactions with the residues Asp-68 and Asp-349, respectively. Also, the arene-cation interactions were observed between the 4-methylphenyl group of **6b** and the residues Arg-439 and Arg-443. It was shown that the residues Thr-215 (bond length: 3.2 Å) and Glu-276 (length: 2.4 Å) formed two hydrogen bonds with **6b**, which was the main interaction between **6b** and α-glucosidase. The interaction between **6p** and α-glucosidase was nearly the same as the compound **6b** (Figure 4B). The main difference was that the 4-methoxyphenyl group of **6p** formed an extra hydrogen bond with the residue Asp-68 (length: 2.4 Å), which made **6p** was more active than **6b** against α-glucosidase (Figure 4B). In addition, the estimated binding energies were −9.5 kcal mol^−1^ for **6b** and −10.1 kcal mol^−1^ for **6p**, respectively, which was consistent with the results of the in vitro α-glucosidase inhibitory activity.

## 3. Experimental Section

### 3.1. General

All starting materials and reagents were purchased from commercial suppliers. TLC was performed on 0.20 mm Silica Gel 60 F_254_ plates (Qingdao Ocean Chemical Factory, Shandong, China). ^1^H- and ^13^C-NMR spectra were recorded were recorded on a Bruker spectrometer (400 MHz) with TMS as an external reference and reported in parts per million.

### 3.2. General Procedures for the Synthesis of ***6a**–**6p***

A mixture of **3** (1.0 mmol) and **5** (1.2 mmol) in EtOH (10 mL) was stirred at reflux for 2 h. After the completion of the reaction, the precipitates that formed were collected by filtration and washed with ethanol (3 × 10 mL) to give the desired products **6a***–***6p**. The spectroscopic and analytical data of compounds are as follows:

*(Z)-3-(2-(4-(4-Bromophenyl)thiazol-2-yl)hydrazono)-5-chloroindolin-2-one* (**6a**). Orange solid, yield 77.5%, ^1^H-NMR (*d*_6_-DMSO, 400 MHz) δ: 6.97 (d, 1H, *J* = 8.4 Hz, ArH), 7.36 (dd, 1H, *J* = 8.4 Hz, 2.0 Hz, ArH), 7.52 (d, 1H, *J* = 2.0 Hz, ArH), 7.61 (d, 2H, *J* = 8.4 Hz, ArH), 7.74 (s, 1H, CH-thiazole), 7.85 (d, 2H, *J* = 8.4 Hz, ArH), 11.36 (s, 1H, NH), 13.33 (s, 1H, NH); ^13^C-NMR (*d*_6_-DMSO, 100 MHz) δ: 108.6, 113.0, 119.8, 121.5, 121.9, 127.1, 128.1, 128.2, 130.3, 131.6, 132.1, 132.2, 133.6, 140.4, 150.4, 163.4, 166.5.

*(Z)-1-(2-Fluorobenzyl)-5-methyl-3-(2-(4-(p-tolyl)thiazol-2-yl)hydrazono) indolin-2-one* (**6b**). Red solid, yield 52.5%, ^1^H-NMR (*d*_6_-DMSO, 400 MHz) δ: 2.33 (s, 6H, CH_3_), 5.06 (s, 2H, CH_2_), 6.93 (d, 1H, *J* = 8.4 Hz, ArH), 7.14–7.19 (m, 2H, ArH), 7.23–7.26 (m, 3H, ArH), 7.32–7.37 (m, 2H, ArH), 7.44 (s, 1H, ArH), 7.58 (s, 1H, ArH), 7.79 (d, 2H, *J* = 8.0 Hz, ArH), 13.22 (s, 1H, NH); ^13^C-NMR (*d*_6_-DMSO, 100 MHz) δ: 21.0, 21.3, 37.4, 106.6, 110.3, 115.9 (d, 1C, *J* = 20.9 Hz), 119.7, 120.6, 122.9, 123.0, 125.2 (d, 1C, *J* = 3.4 Hz), 126.0, 126.1, 129.7, 129.9, 130.0, 130.1, 130.3 (d, 1C, *J* = 8.1 Hz), 131.2, 131.4, 131.8, 132.9, 137.8, 139.7, 151.7, 159.4 (d, 1C, *J* = 243.9 Hz), 161.8, 166.3; MS (ESI, *m*/*z*): 457.11 [M + H]^+^.

*(Z)-1-(2-Fluorobenzyl)-3-(2-(4-(4-methoxyphenyl)thiazol-2-yl)hydrazono)-5-methylindolin-2-one* (**6c**). Red solid, yield 77.2%, ^1^H-NMR (*d*_6_-DMSO, 400 MHz) δ: 2.32 (s, 3H, CH_3_), 3.79 (s, 3H, OCH_3_), 5.06 (s, 2H, CH_2_), 6.92 (d, 1H, *J* = 8.0 Hz, ArH), 6.98 (d, 2H, *J* = 8.0 Hz, ArH), 7.13–7.18 (m, 2H, ArH), 7.22–7.26 (m, 1H, ArH), 7.32–7.37 (m, 2H, ArH), 7.43 (s, 1H, ArH), 7.48 (s, 1H, CH-thiazole), 7.83 (d, 2H, *J* = 8.0 Hz, ArH), 13.21 (s, 1H, NH); ^13^C-NMR (*d*_6_-DMSO, 100 MHz) δ: 21.0, 37.4, 55.6, 105.3, 110.3, 114.5, 114.7, 115.9 (d, 1C, *J* = 20.9 Hz), 119.7, 120.5, 122.9, 123.0, 125.2 (d, 1C, *J* = 3.5 Hz), 127.3, 127.6, 130.0, 130.1, 130.3 (d, 1C, *J* = 8.1 Hz), 131.2, 131.3, 132.8, 139.7, 151.5, 159.4 (d, 1C, *J* = 243.9 Hz), 159.6, 161.7; MS (ESI, *m*/*z*): 473.09 [M + H]^+^.

*(Z)-1-Methyl-3-(2-(4-(p-tolyl)thiazol-2-yl)hydrazono)indolin-2-one* (**6d**). Red solid, yield 75.4%, ^1^H-NMR (*d*_6_-DMSO, 400 MHz) δ: 2.33(s, 3H, ArCH_3_), 3.27 (s, 3H, NCH_3_), 7.15–7.19 (m, 2H, ArH), 7.23 (d, 2H, *J* = 8.0 Hz, ArH), 7.44 (t, 1H, *J* = 8.0 Hz, ArH), 7.57 (s, 1H, CH-thiazole), 7.57 (d, 1H, *J* = 8.0 Hz, ArH), 7.79 (d, 2H, *J* = 8.0 Hz, ArH), 13.26 (s, 1H, NH); ^13^C-NMR (*d*_6_-DMSO, 100 MHz) δ: 21.3, 26.2, 106.4, 110.2, 119.5, 119.9, 123.3, 126.1, 129.7, 130.8, 131.6, 131.8, 137.8, 143.0, 151.6, 161.8, 166.3.

*(Z)-3-(2-(4-(4-Fluorophenyl)thiazol-2-yl)hydrazono)-1-methylindolin-2-one* (**6e**). Orange solid, yield 70.3%, ^1^H-NMR (*d*_6_-DMSO, 400 MHz) δ: 3.27 (s, 3H, NCH_3_), 7.15–7.18 (m, 2H, ArH), 7.26 (t, 2H, *J* = 8.8 Hz, ArH), 7.44 (t, 1H, *J* = 8.0 Hz, ArH), 7.57 (d, 1H, *J* = 8.0 Hz, ArH), 7.62 (s, 1H, CH-thiazole), 7.93 (dd, 2H, *J* = 8.8 Hz, 5.6 Hz, ArH), 13.25 (s, 1H, NH); ^13^C-NMR (*d*_6_-DMSO, 100 MHz) δ: 26.2, 107.1, 110.3, 115.9 (d, 1C, *J* = 21.5 Hz), 119.5, 120.0, 123.4, 128.2 (d, 1C, *J* = 8.1 Hz), 130.9, 131.0 (d, 1C, *J* = 2.9 Hz), 131.1, 131.9, 143.1, 150.5, 161.1 (d, 1C, *J* = 243.7 Hz), 161.8, 166.6.

*(Z)-3-(2-(4-(4-Methoxyphenyl)thiazol-2-yl)hydrazono)-1-methylindolin-2-one* (**6f**). Red solid, yield 69.9%, ^1^H-NMR (*d*_6_-DMSO, 400 MHz) δ: 3.27 (s, 3H, NCH_3_), 3.80 (s, 3H, OCH_3_), 6.98 (d, 2H, *J* = 8.8 Hz, ArH), 7.15–7.19 (m, 2H, ArH), 7.44 (t, 1H, *J* = 8.0 Hz, ArH), 7.48 (s, 1H, CH-thiazole), 7.58 (d, 1H, *J* = 7.2 Hz, ArH), 7.83 (d, 2H, *J* = 8.8 Hz, ArH), 13.26 (s, 1H, NH); ^13^C-NMR (*d*_6_-DMSO, 100 MHz) δ: 26.2, 55.6, 105.2, 110.3, 114.5, 114.7, 119.5, 119.9, 123.4, 127.3, 127.5, 127.6, 130.8, 131.6, 143.0, 151.5, 159.6, 161.8, 166.3.

*(Z)-3-(2-(4-(4-Chlorophenyl)thiazol-2-yl)hydrazono)indolin-2-one* (**6g**). Orange solid, yield 66.9%, ^1^H-NMR (*d*_6_-DMSO, 400 MHz) δ: 6.96 (d, 1H, *J* = 8.8 Hz, ArH), 7.10 (t, 1H, *J* = 8.8 Hz, ArH), 7.35 (t, 1H, *J* = 8.8 Hz, ArH), 7.47 (d, 2H, *J* = 8.8 Hz, ArH), 7.54 (d, 1H, *J* = 8.8 Hz, ArH), 7.70 (s, 1H, CH-thiazole), 7.92 (d, 2H, *J* = 8.8 Hz, ArH), 11.26 (s, 1H, NH-indolin-2-one),13.35 (s, 1H, NH); ^13^C-NMR (*d*_6_-DMSO, 100 MHz) *δ*: 108.0, 111.5, 120.2, 120.3, 122.9, 127.9, 129.1, 131.0, 132.7, 132.9, 133.3, 141.8, 150.3, 163.6, 166.7.

*(Z)-3-(2-(4-(4-Bromophenyl)thiazol-2-yl)hydrazono)-5,7-dimethylindolin-2-one* (**6h**). Orange solid, yield 64.4%, ^1^H-NMR (*d*_6_-DMSO, 400 MHz) δ: 2.21 (s, 3H, ArCH_3_), 2.29 (s, 3H, ArCH_3_), 6.99 (s, 1H, ArH), 7.19 (s, 1H, ArH), 7.61 (d, 2H, *J* = 8.8 Hz, ArH), 7.71 (s, 1H, CH-thiazole), 7.85 (d, 2H, *J* = 8.8 Hz, ArH), 11.22 (s, 1H, NH-indolin-2-one), 13.40 (s, 1H, NH); ^13^C-NMR (*d*_6_-DMSO, 100 MHz) δ: 16.3, 20.9, 107.9, 118.1, 119.9, 120.6, 121.4, 128.2, 131.9, 132.0, 132.9, 133.2, 133.7, 138.2, 150.3, 164.2, 166.7.

*(Z)-3-(2-(4-(4-Hydroxyphenyl)thiazol-2-yl)hydrazono)-5-methylindolin-2-one* (**6i**). Orange solid, yield 52.4%, ^1^H-NMR (*d*_6_-DMSO, 400 MHz) δ: 2.33 (s, 3H, ArCH_3_), 6.80 (d, 2H, *J* = 8.8 Hz, ArH), 6.85 (d, 1H, *J* = 8.0 Hz, ArH), 7.15 (d, 1H, *J* = 8.0 Hz, ArH), 7.36 (s, 2H, CH-thiazole and ArH), 7.71 (d, 2H, *J* = 8.8 Hz, ArH), 11.16 (s, 1H, NH-indolin-2-one), 13.35 (s, 1H, NH); ^13^C-NMR (*d*_6_-DMSO, 100 MHz) δ: 21.0, 104.2, 111.3, 115.6, 115.9, 120.2, 120.6, 125.6, 127.6, 129.9, 131.4, 131.9, 132.7, 139.5, 151.5, 157.9, 163.7, 166.3.

*(Z)-3-(2-(4-(4-Fluorophenyl)thiazol-2-yl)hydrazono)-5,7-dimethylindolin-2-one* (**6j**). Orange solid, yield 59.6%, ^1^H-NMR (*d*_6_-DMSO, 400 MHz) δ: 2.21 (s, 3H, ArCH_3_), 2.29 (s, 3H, ArCH_3_), 6.99 (s, 1H, ArH), 7.19 (s, 1H, ArH), 7.26 (t, 2H, *J* = 8.8 Hz), 7.61 (s, 1H, CH-thiazole), 7.93 (d, 2H, *J* = 8.8 Hz, 5.6 Hz, ArH), 11.22 (s, 1H, NH-indolin-2-one), 13.39 (s, 1H, NH); ^13^C-NMR (*d*_6_-DMSO, 100 MHz) δ: 16.3, 20.9, 106.8, 115.8 (d, 1C, *J* = 21.5 Hz), 118.1, 119.9, 120.6, 128.2 (d, 1C, *J* = 8.1 Hz), 131.1 (d, 1C, *J* = 2.7 Hz), 131.8, 132.8, 133.1, 138.2, 150.5, 161.1 (d, 1C, *J* = 243.7 Hz), 164.2, 166.6.

*(Z)-5,7-Dimethyl-3-(2-(4-(p-tolyl)thiazol-2-yl)hydrazono)indolin-2-one* (**6k**). Orange solid, yield 62.1%, ^1^H-NMR (*d*_6_-DMSO, 400 MHz) δ: 2.21 (s, 3H, ArCH_3_), 2.29 (s, 3H, ArCH_3_), 2.34 (s, 3H, ArCH_3_), 6.99 (s, 1H, ArH), 7.20 (s, 1H, ArH), 7.23 (d, 2H, *J* = 8.0 Hz, ArH), 7.55 (s, 1H, CH-thiazole), 7.79 (d, 2H, *J* = 8.0 Hz, ArH),11.22 (s, 1H, NH-indolin-2-one), 13.39 (s, 1H, NH); ^13^C-NMR (*d*_6_-DMSO, 100 MHz) δ: 16.3, 20.9, 21.3, 106.2, 118.1, 119.9, 120.6, 126.1, 129.7, 131.9, 132.8, 133.0, 137.7, 138.2, 151.6, 164.2, 166.4.

*(Z)-5-Fluoro-3-(2-(4-(p-tolyl)thiazol-2-yl)hydrazono)indolin-2-one* (**6l**). Orange solid, yield 60.8%, ^1^H-NMR (*d*_6_-DMSO, 400 MHz) δ: 2.24 (s, 3H, ArCH_3_), 6.96 (dd, 1H, *J* = 8.4 Hz, 4.4 Hz, ArH), 7.19 (dt, 1H, *J* = 8.4 Hz, 2.4 Hz, ArH), 7.23 (d, 2H, *J* = 8.0 Hz, ArH), 7.35 (dd, 1H, *J* = 8.4 Hz, 2.4 Hz, ArH), 7.59 (s, 1H, CH-thiazole), 7.79 (d, 2H, *J* = 8.0 Hz, ArH), 11.28 (s, 1H, NH-indolin-2-one), 13.39 (s, 1H, NH); ^13^C-NMR (*d*_6_-DMSO, 100 MHz) δ: 21.3, 106.7, 107.1, 107.4, 112.5 112.6, 117.0 (d, 1C, *J* = 24.5 Hz), 121.4 (d, 1C, *J* = 8.9 Hz), 125.9, 126.1, 129.7, 129.9, 131.7, 131.9 (d, 1C, *J* = 3.1 Hz), 137.8, 137.9, 151.7, 157.6 (d, 1C, *J* = 236.4 Hz), 163.8, 166.1.

*(Z)-5-Fluoro-3-(2-(4-(4-fluorophenyl)thiazol-2-yl)hydrazono)indolin-2-one* (**6m**). Orange solid, yield 57.7%, ^1^H-NMR (*d*_6_-DMSO, 400 MHz) δ: 6.95 (dd, 1H, *J* = 8.4 Hz, 4.4 Hz, ArH), 7.19 (dt, 1H, *J* = 8.4 Hz, 2.4 Hz, ArH), 7.25 (d, 2H, *J* = 8.8 Hz, ArH), 7.35 (dd, 1H, *J* = 8.4 Hz, 2.4 Hz, ArH), 7.66 (s, 1H, CH-thiazole), 7.94 (dd, 2H, *J* = 8.8 Hz, 5.6 Hz, ArH), 11.28 (s, 1H, NH-indolin-2-one), 13.39 (s, 1H, NH); ^13^C-NMR (*d*_6_-DMSO, 100 MHz) δ: 107.2, 107.4, 107.4, 112.5, 112.6, 115.9 (d, 1C, *J* = 21.4 Hz), 116.2 (d, 1C, *J* = 21.5 Hz), 117.1, 117.3, 121.4, 121.5, 128.2, 128.3, 128.4, 131.0 (d, 1C, *J* = 2.9 Hz), 132.0 (d, 1C, *J* = 3.6 Hz), 138.0, 150.5, 157.6 (d, 1C, *J* = 236.3 Hz), 161.1 (d, 1C, *J* = 243.6 Hz), 163.8, 166.3.

*(Z)-3-(2-(4-(4-Fluorophenyl)thiazol-2-yl)hydrazono)indolin-2-one* (**6n**). Orange solid, yield 78.4%, ^1^H-NMR (*d*_6_-DMSO, 400 MHz) *δ:* 6.97 (d, 1H, *J* = 8.0 Hz, ArH), 7.10 (t, 1H, *J* = 8.0 Hz, ArH), 7.26 (d, 2H, *J* = 8.8 Hz, ArH), 7.36 (t, 1H, *J* = 8.0 Hz, ArH), 7.54 (d, 1H, *J* = 8.0 Hz, ArH), 7.62 (s, 1H, CH-thiazole), 7.94 (dd, 2H, *J* = 8.8 Hz, 5.6 Hz, ArH), 11.27 (s, 1H, NH-indolin-2-one), 13.35 (s, 1H, NH); ^13^C-NMR (*d*_6_-DMSO, 100 MHz) δ: 117.0, 111.5, 115.9 (d, 1C, *J* = 21.4 Hz), 120.2, 120.3, 122.9, 128.2 (d, 1C, *J* = 8.1 Hz), 130.9, 131.1 (d, 1C, *J* = 2.9 Hz), 132.6, 141.8, 150.5, 161.1(d, 1C, *J* = 243.6 Hz), 163.6, 166.6.

*(Z)-3-(2-(4-(p-Tolyl)thiazol-2-yl)hydrazono)indolin-2-one* (**6o**). Red solid, yield 73.3%, ^1^H-NMR (*d*_6_-DMSO, 400 MHz) δ: 2.34 (s, 3H, ArCH_3_), 6.97 (d, 1H, *J* = 8.0 Hz, ArH), 7.10 (t, 1H, *J* = 8.0 Hz, ArH), 7.23 (d, 2H, *J* = 8.0 Hz, ArH), 7.35 (t, 1H, *J* = 8.0 Hz, ArH), 7.54–7.56 (m, 2H, CH-thiazole and ArH), 7.79 (d, 2H, *J* = 8.4 Hz, ArH), 11.27 (s, 1H, NH-indolin-2-one), 13.35 (s, 1H, NH); ^13^C-NMR (*d*_6_-DMSO, 100 MHz) δ: 21.3, 106.3, 111.5, 120.2, 120.3, 122.9, 126.1, 129.7, 129.8, 130.9, 131.8, 132.5, 137.8, 141.7, 151.6, 163.7, 166.4.

*(Z)-1-(2-Fluorobenzyl)-3-(2-(4-(4-hydroxyphenyl)thiazol-2-yl)hydrazono)-5-methylindolin-2-one* (**6p**). Red solid, yield 53.1%, ^1^H-NMR (*d*_6_-DMSO, 400 MHz) δ: 2.33 (s, 3H, ArCH_3_), 5.07 (s, 2H, ArCH_2_), 6.81 (d, 2H, *J* = 7.2 Hz, ArH), 6.93 (d, 1H, *J* = 7.2 Hz, ArH), 7.12–7.25 (m, 3H, ArH), 7.36–7.44 (m, 3H), 7.72 (d, 2H, *J* = 7.2 Hz, ArH), 9.62 (s, 1H, OH), 13.21 (s, 1H, NH); ^13^C-NMR (*d*_6_-DMSO, 100 MHz) δ: 21.0, 37.4, 104.4, 110.3, 115.9, 115.9 (d, 1C, *J* = 23.0 Hz), 119.8, 120.5, 122.9, 123.0, 125.2, 125.8, 127.6, 130.0, 130.2 (d, 1C, *J* = 8.1 Hz), 131.1, 132.8, 139.6, 151.9, 157.9, 159.4 (d, 1C, *J* = 237.9 Hz), 166.0; MS (ESI, *m*/*z*): 459.06 [M + H]^+^.

### 3.3. In Vitro Assay of α-Glucosidase Inhibitory Activity

According to the literature procedure [36], α-Glucosidase inhibitory activity was assayed by using 0.1 M phosphate buffer (pH 6.8) at 37 °C. The enzyme (0.1 U/mL) in phosphate buffer saline was incubated with various concentrations of test compounds at 37 °C for 15 min. Then 1.25 mM *p*-nitrophenyl α-d-glucopyranoside was added to the mixture as a substrate, after further incubation at 37 °C for 30 min. The absorbance was measured spectrophotometrically at 405 nm. The sample solution was replaced by DMSO as a control. Acarbose was used as a positive control. All experiments were carried out in triplicates. The % inhibition has been obtained using the formula: inhibition (%) = (1 − ∆Asample/∆Acontrol) × 100%. IC_50_ value is defined as a concentration of samples inhibiting 50% of α-glucosidase activity under the stated assay conditions. 

### 3.4. Molecular Docking

Molecular docking studies were performed to investigate the binding mode between the compounds **6b**, **6i**, **6p**, and α-glucosidase using Autodock vina 1.1.2 [38]. The 3D structures of **6i** and **6p** were obtained by ChemBioDraw Ultra 14.0 and ChemBio3D Ultra 14.0 softwares. The AutoDockTools 1.5.6 package was employed to generate the docking input files [39,40]. The search grid of α-glucosidase was identified as center_x: −19.676, center_y: −7.243, and center_z: −21.469 with dimensions size_x: 15, size_y: 15, and size_z: 15. The value of exhaustiveness was set to 20. For Vina docking, the default parameters were used if it was not mentioned. The best-scoring poses as judged by the Vina docking score were chosen and visually analyzed using PyMOL 1.7.6 software (Schrödinger^®^, New York, NY, USA) (http://www.pymol.org/).

## 4. Conclusions

In conclusion, we designed and synthesized a novel series of α-glucosidase inhibitor based on the molecular hybrid-based approaches. All the target compounds displayed potent to moderate α-glucosidase inhibitory activity with IC_50_ ranging from 5.36 ± 0.13 to 35.76 ± 0.31 μm as compared to the standard drug acarbose (IC_50_ = 817.38 ± 6.27 μm). Among the series, compound **6p** bearing a hydroxyl group at the 4-position of the right phenyl and 2-fluorobenzyl substituent at the N1-positions of the 5-methylisatin displayed the highest inhibitory activity with an IC_50_ value of 5.36 ± 0.13 μm. Furthermore, molecular docking studies revealed the existence of hydrophobic interaction, CH-π interaction, arene-anion interaction, arene-cation interaction, and hydrogen bond between these compounds and α-glucosidase enzyme.

## Figures and Tables

**Table 1:** α-Glucosidase inhibitory activity of novel isatin-thiazole derivatives (**6a**–**6p**).

Compound	R_1_	R_2_	R_3_	IC_50_ (μm) ^a^
**6a**	5-Cl	H	Br	6.87 ± 0.14
**6b**	5-Me	2-F-benzyl	Me	35.76 ± 0.31
**6c**	5-Me	2-F-benzyl	MeO	10.34 ± 0.17
**6d**	H	Me	Me	32.17 ± 0.29
**6e**	H	Me	F	33.20 ± 0.24
**6f**	H	Me	MeO	24.17 ± 0.28
**6g**	H	H	Cl	5.98 ± 0.12
**6h**	5.7-Me_2_	H	Br	6.12 ± 0.15
**6i**	5-Me	H	OH	7.51 ± 0.17
**6j**	5.7-Me_2_	H	F	6.51 ± 0.13
**6k**	5.7-Me_2_	H	Me	15.68 ± 0.24
**6l**	5-F	H	Me	8.33 ± 0.18
**6m**	5-F	H	F	7.17 ± 0.15
**6n**	H	H	F	6.36 ± 0.12
**6o**	H	H	Me	11.78 ± 0.21
**6p**	5-Me	2-F-benzyl	OH	5.36 ± 0.13
**Acarbose**				817.38 ± 6.27

^a^ Acarbose is standard for α-glucosidase inhibition activity.

## Supplementary Materials

Supplementary materials are available online.
