# Supplementary material for: Synthesis, Biological Evaluation, and Molecular Docking Studies of Novel Isatin-Thiazole Derivatives as α-Glucosidase Inhibitors
Source: Molecules. 2017 Apr 20;22(4):659. doi: 10.3390/molecules22040659 (PMC6154535; doi:10.3390/molecules22040659)
Supplement: Supplementary file 1 [file molecules-22-00659-s001.pdf]

Supplemental Materials

Synthesis, biological evaluation and molecular docking studies of novel isatin-thiazole derivatives as α-glucosidase inhibitors

ZhenZhen Xie, Guangcheng Wang*, Jing Wang, Ming Chen, Yaping Peng, Luyao Li, Bing Deng, Shan Chen, Wenbiao Li

College of Chemistry and Chemical Engineering, Hunan Engineering Laboratory for Analyse and Drugs Development of Ethnomedicine in Wuling Mountains, Jishou University, Jishou 416000, PR China

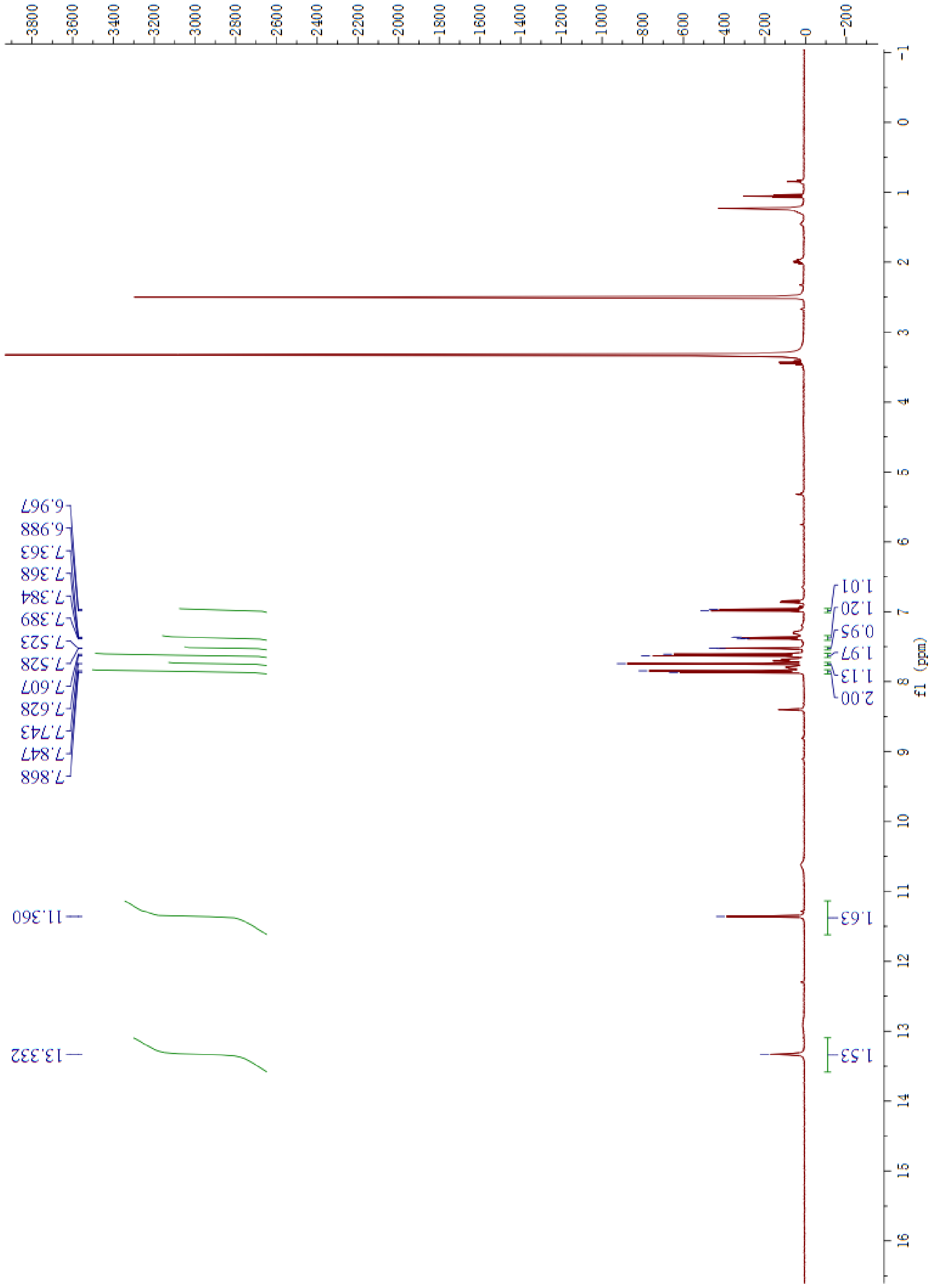

Figure S 1: ^{1}H NMR of Compound **6a**

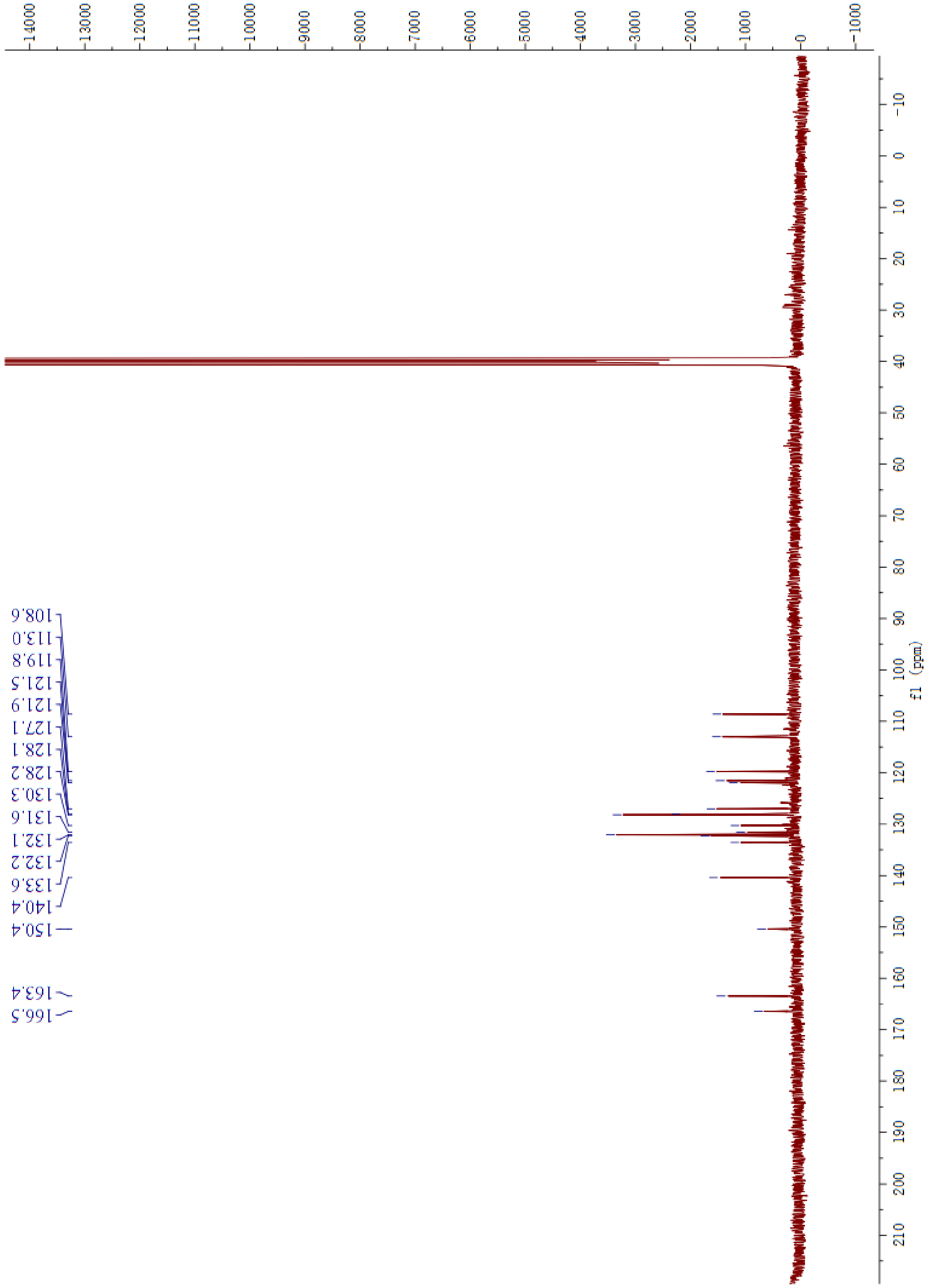

Figure S 2: ^{13}C NMR of Compound **6a**

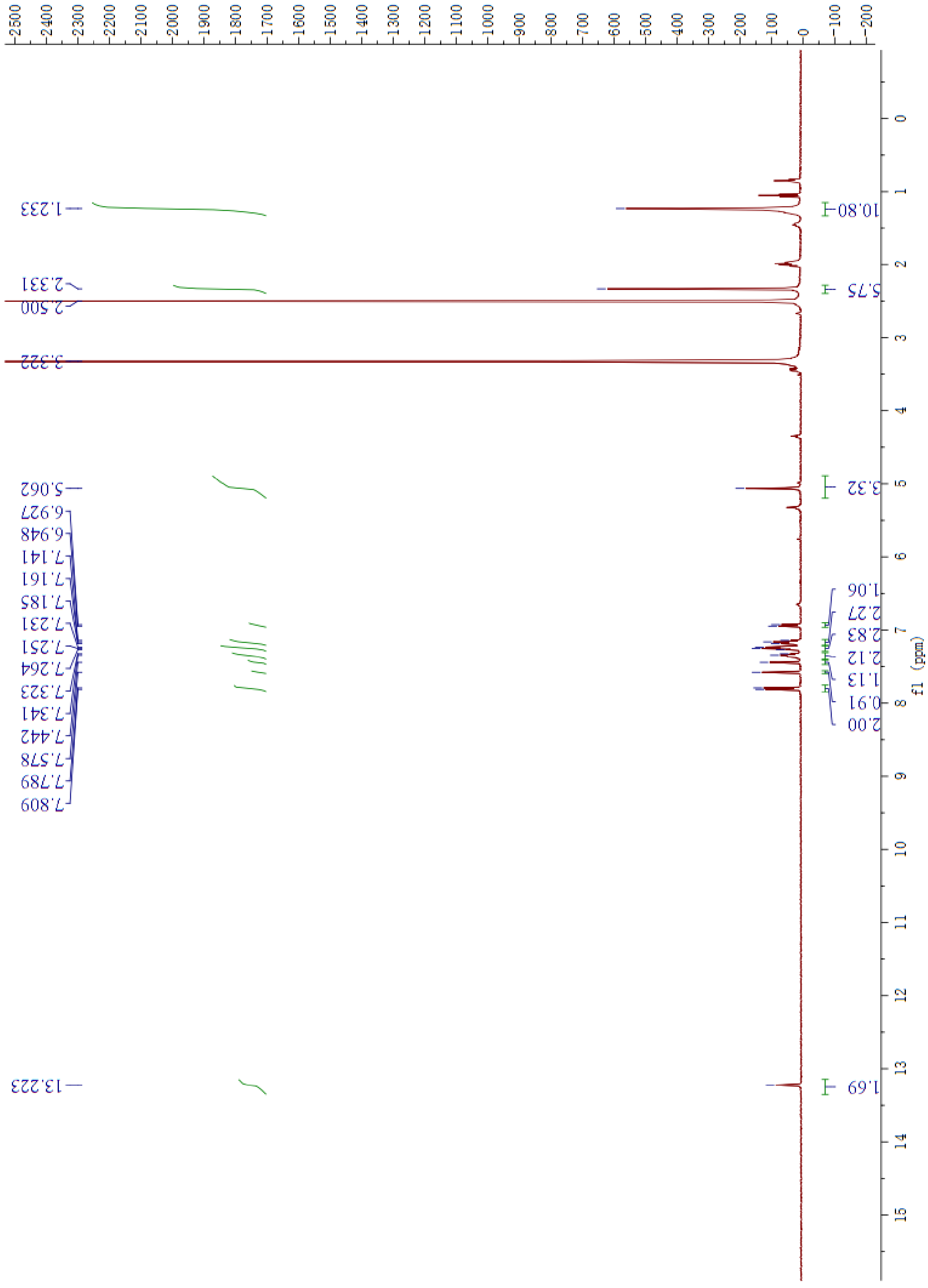

Figure S 3: ^{1}H NMR of Compound **6b**

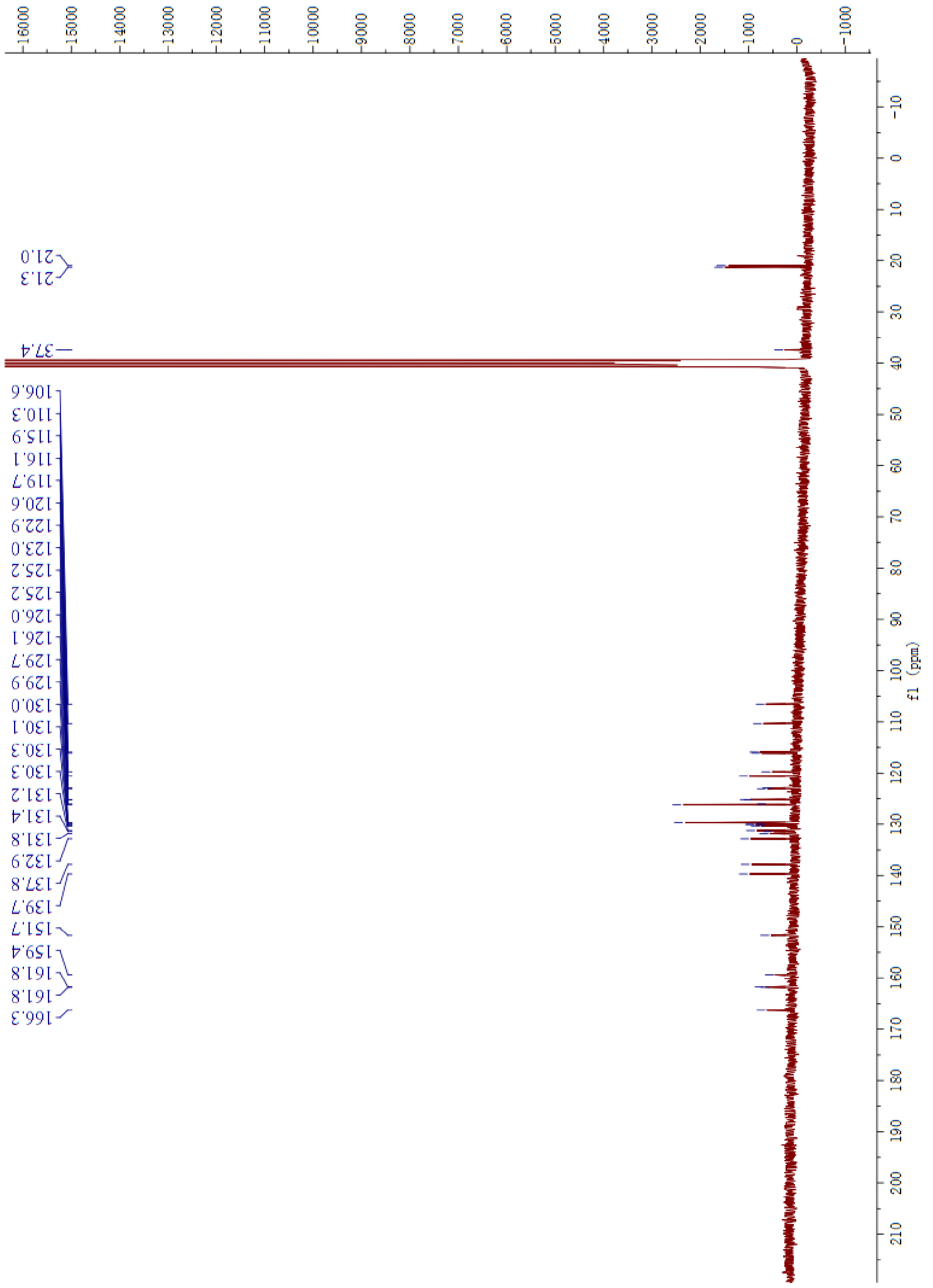

Figure S 4: ^{13}C NMR of Compound **6b**

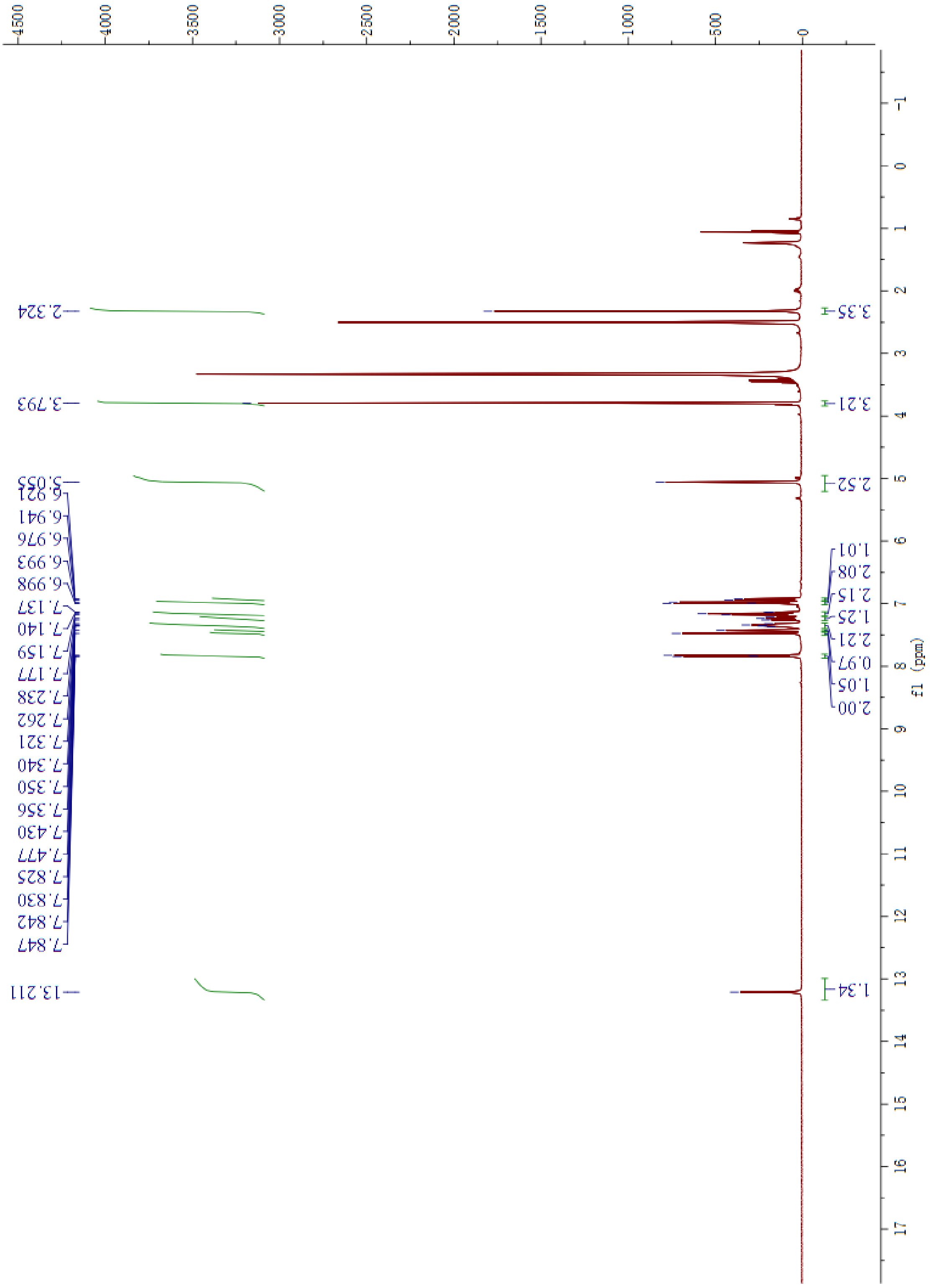

Figure S 5: ^{1}H NMR of Compound **6c**

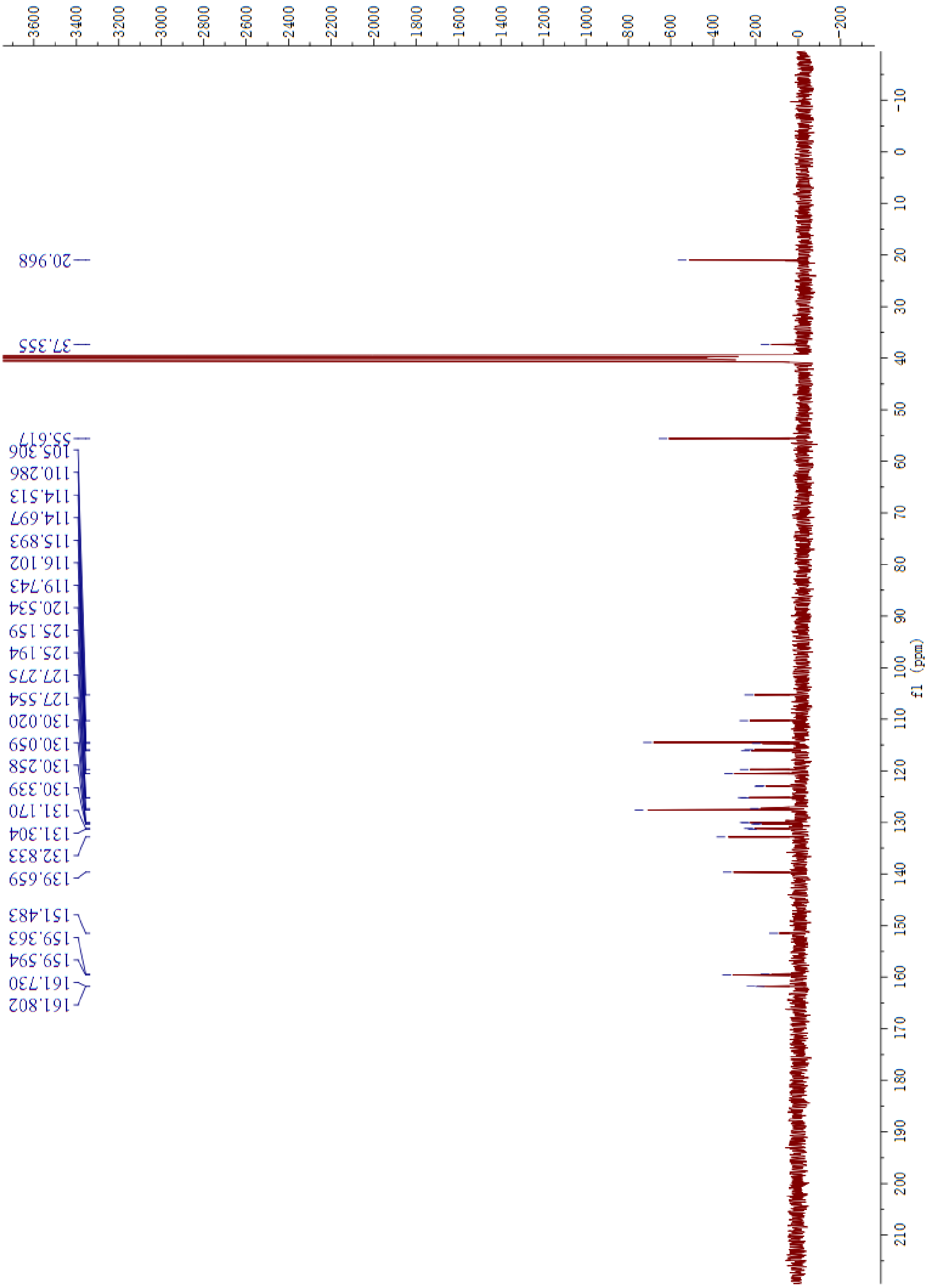

Figure S 6: ^{13}C NMR of Compound **6c**

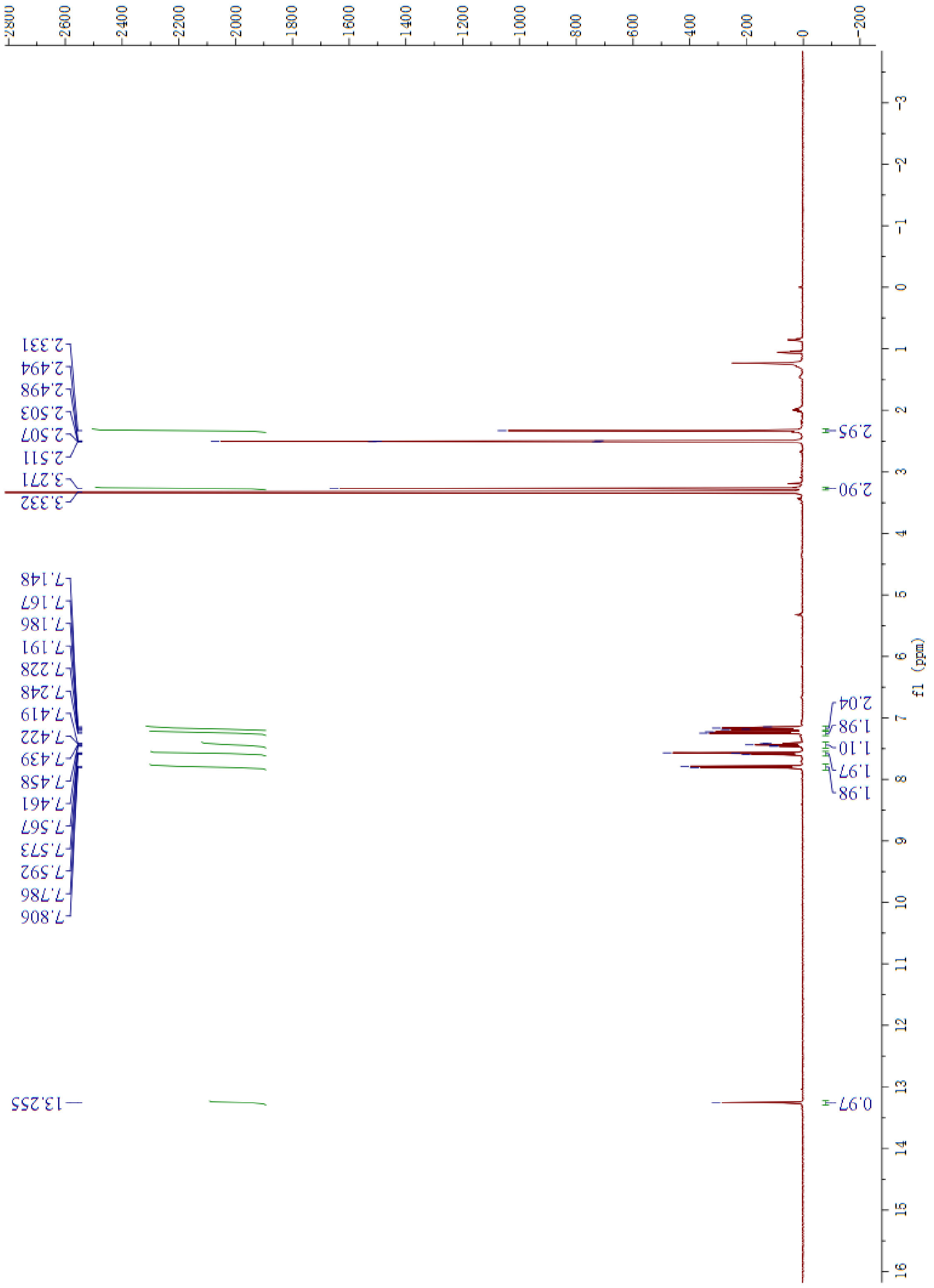

Figure S 7: ^{1}H NMR of Compound **6d**

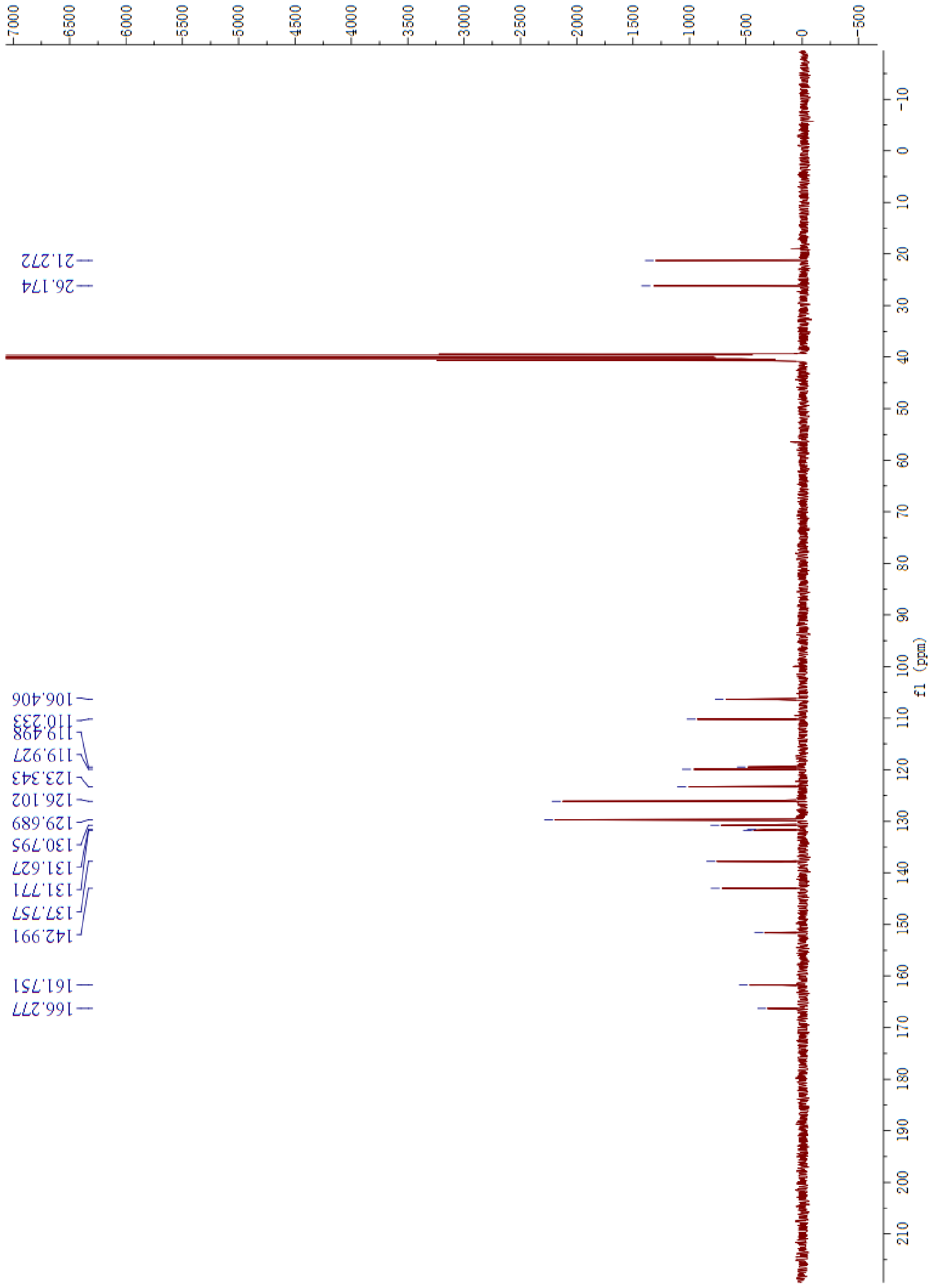

Figure S 8: ^{13}C NMR of Compound **6d**

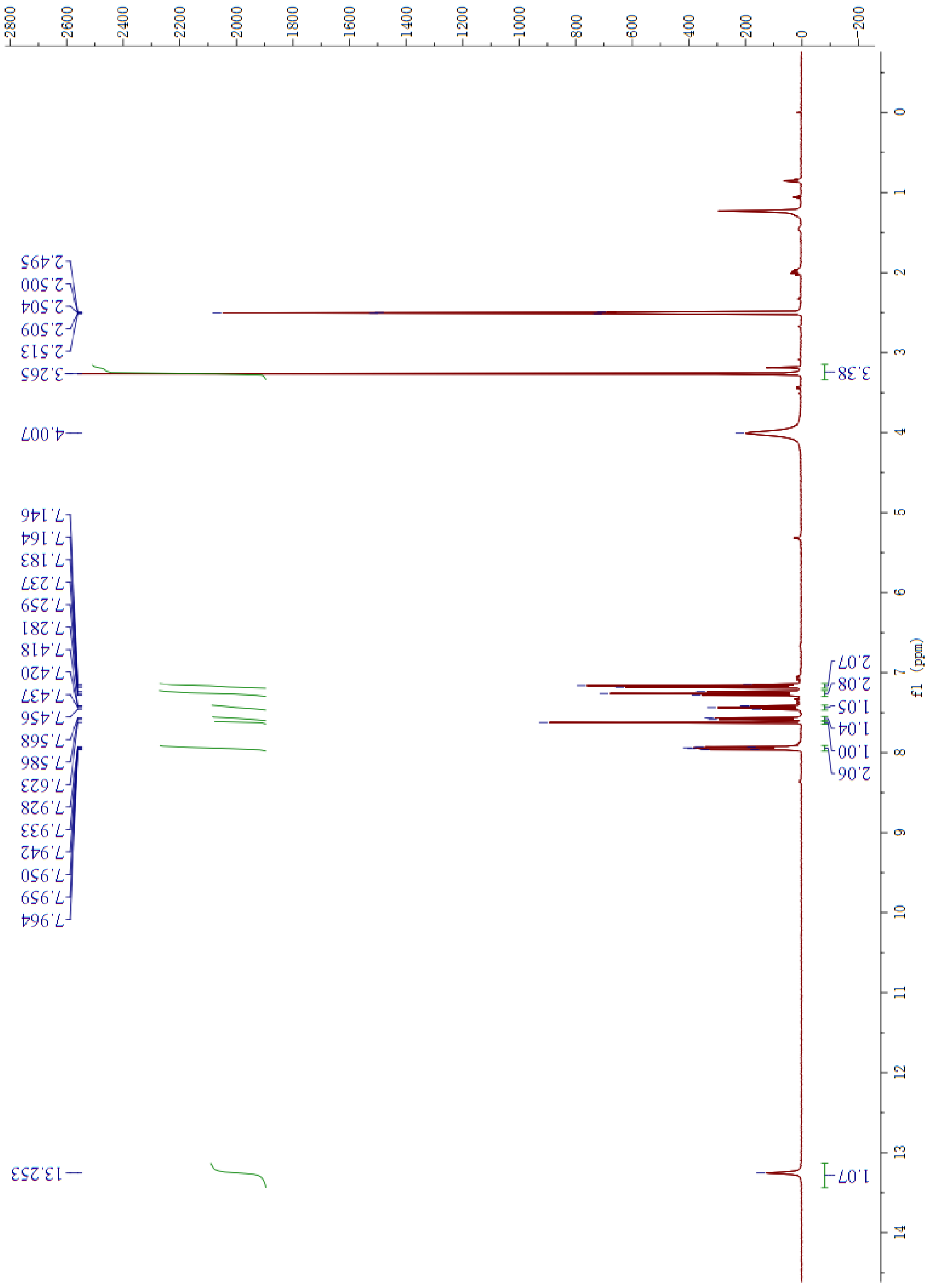

Figure S 9: ^{1}H NMR of Compound **6e**

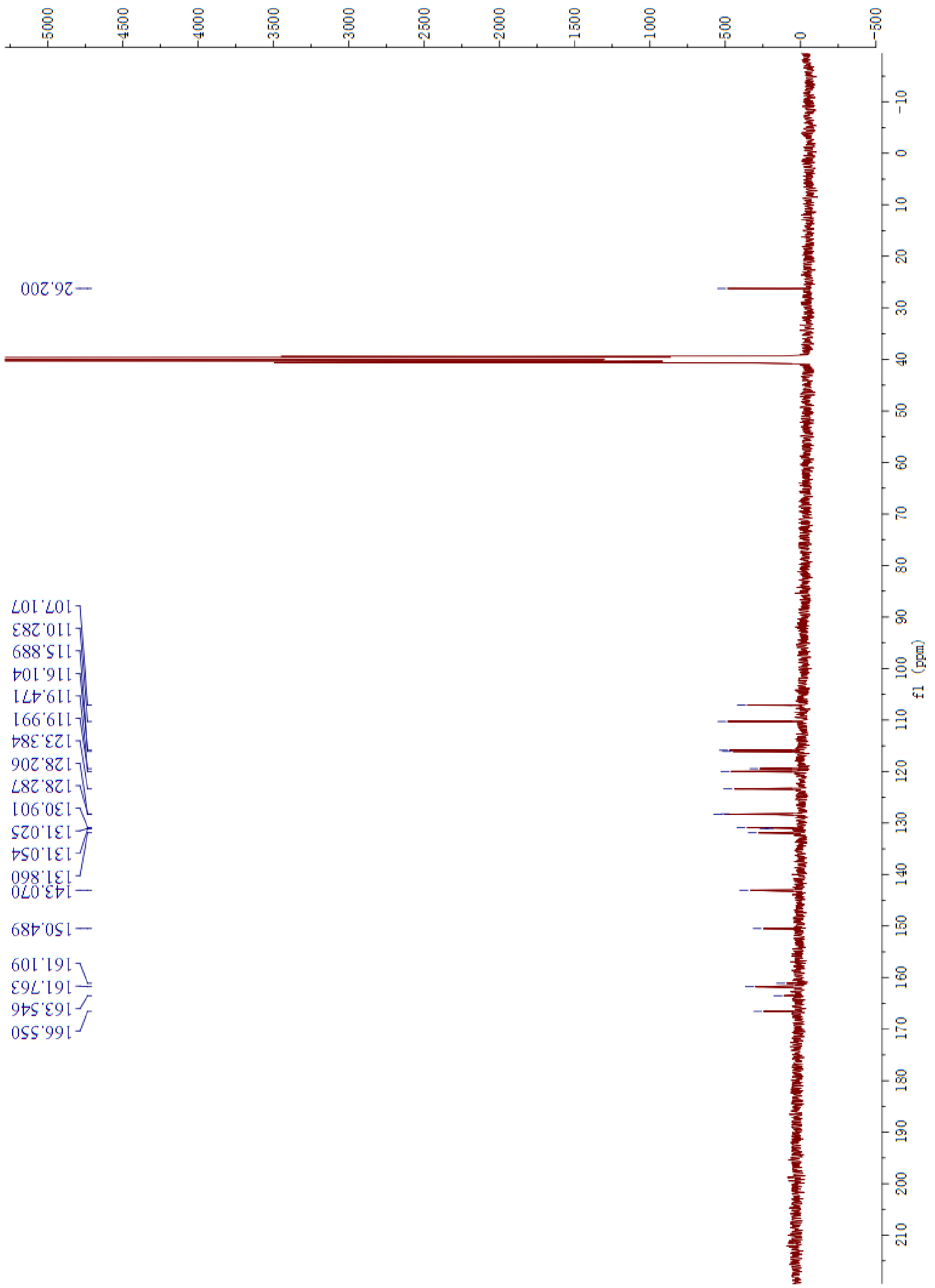

Figure S 10: ^{13}C NMR of Compound **6e**

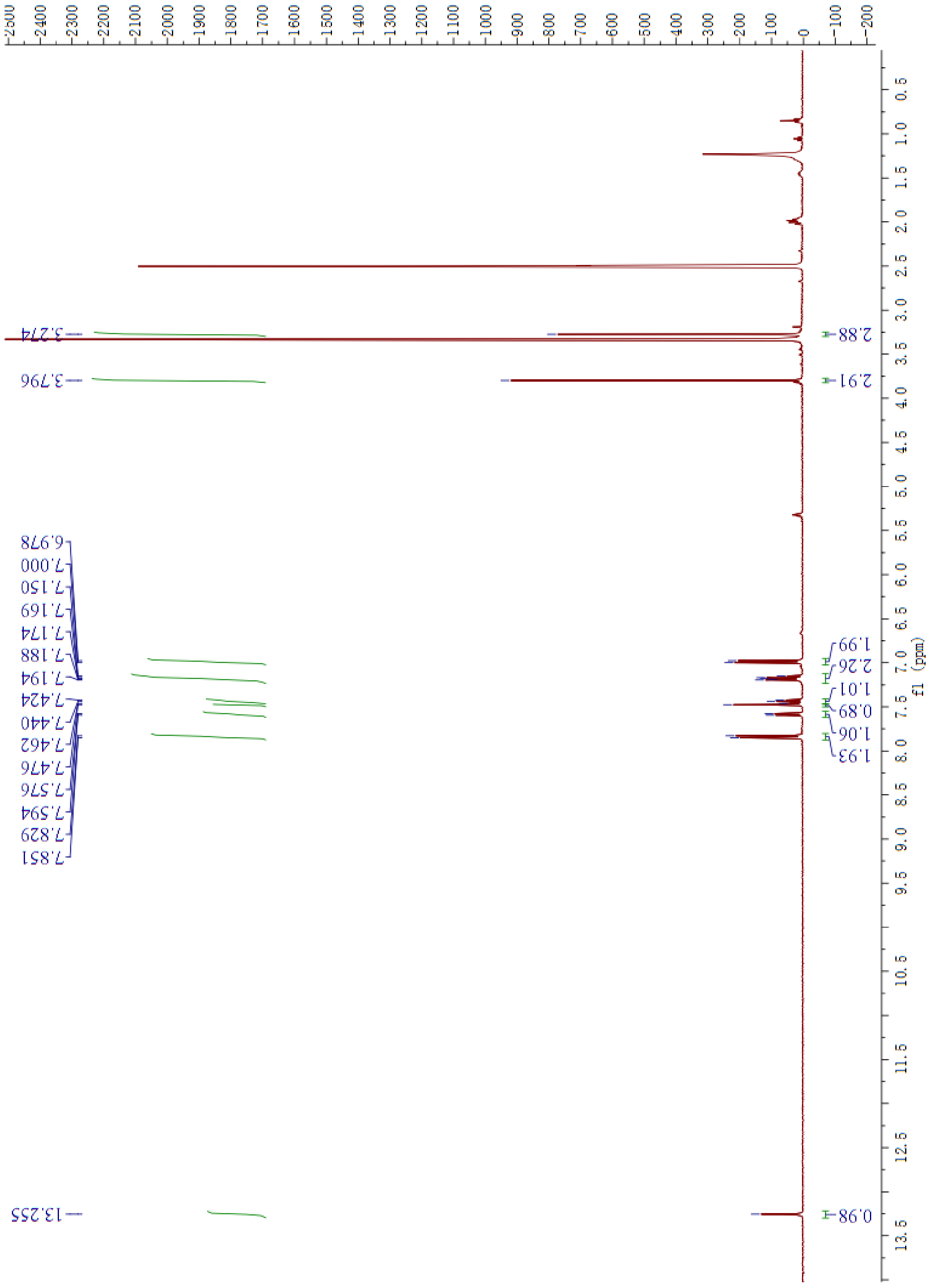

Figure S 11: ^{1}H NMR of Compound **6f**

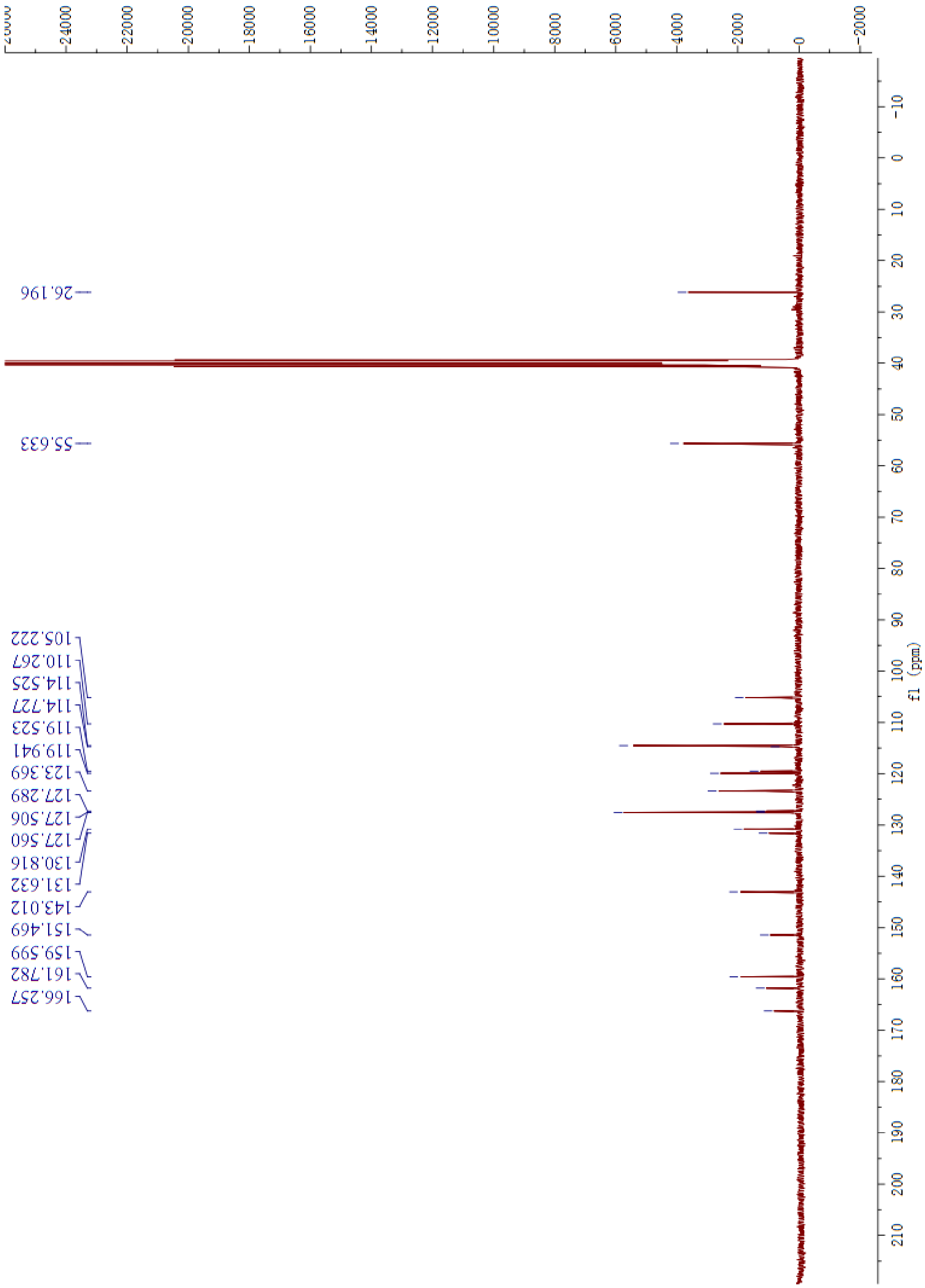

Figure S 12: ^{13}C NMR of Compound **6f**

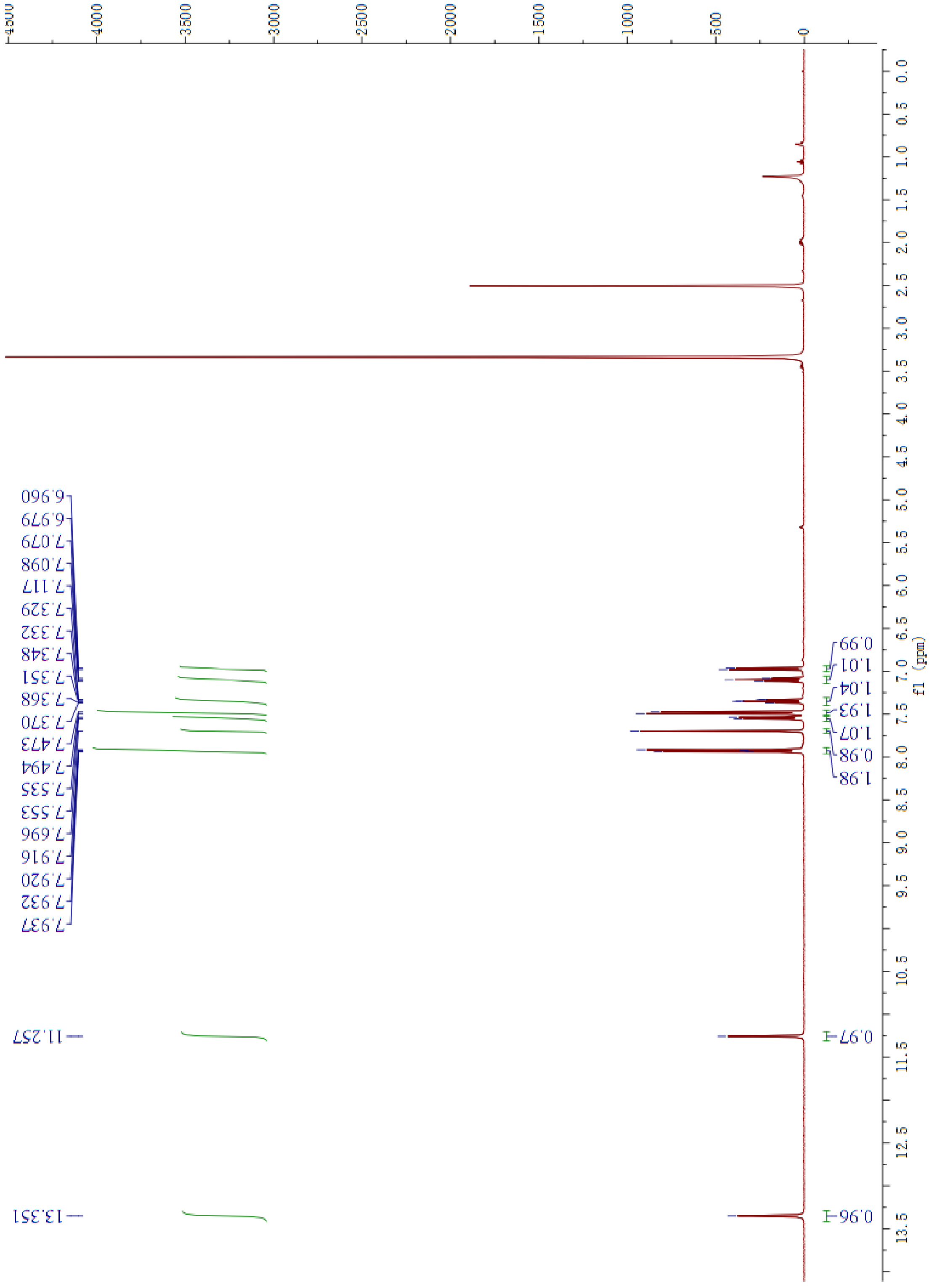

Figure S 13: ^{1}H NMR of Compound **6g**

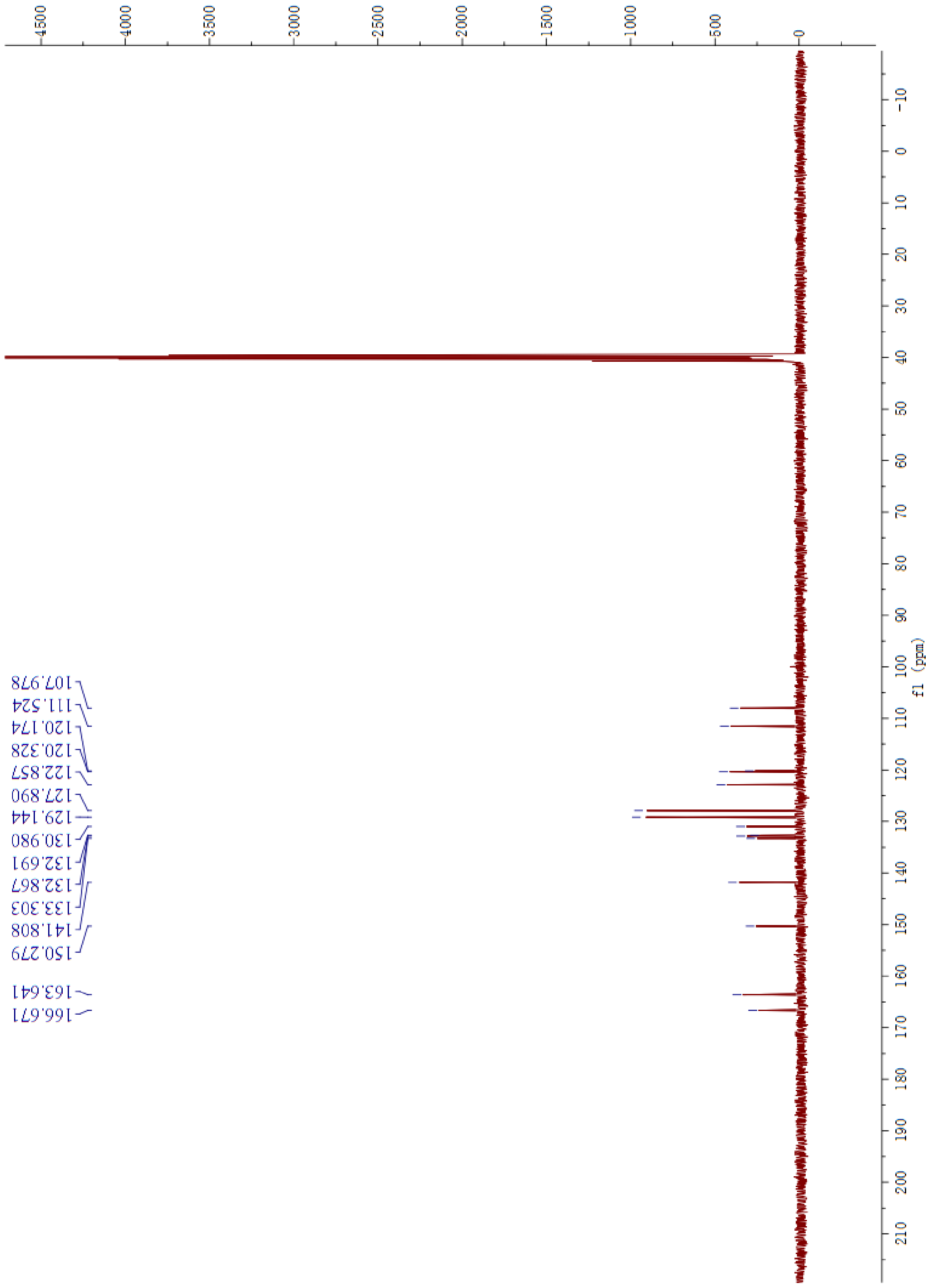

Figure S 14: ^{13}C NMR of Compound **6g**

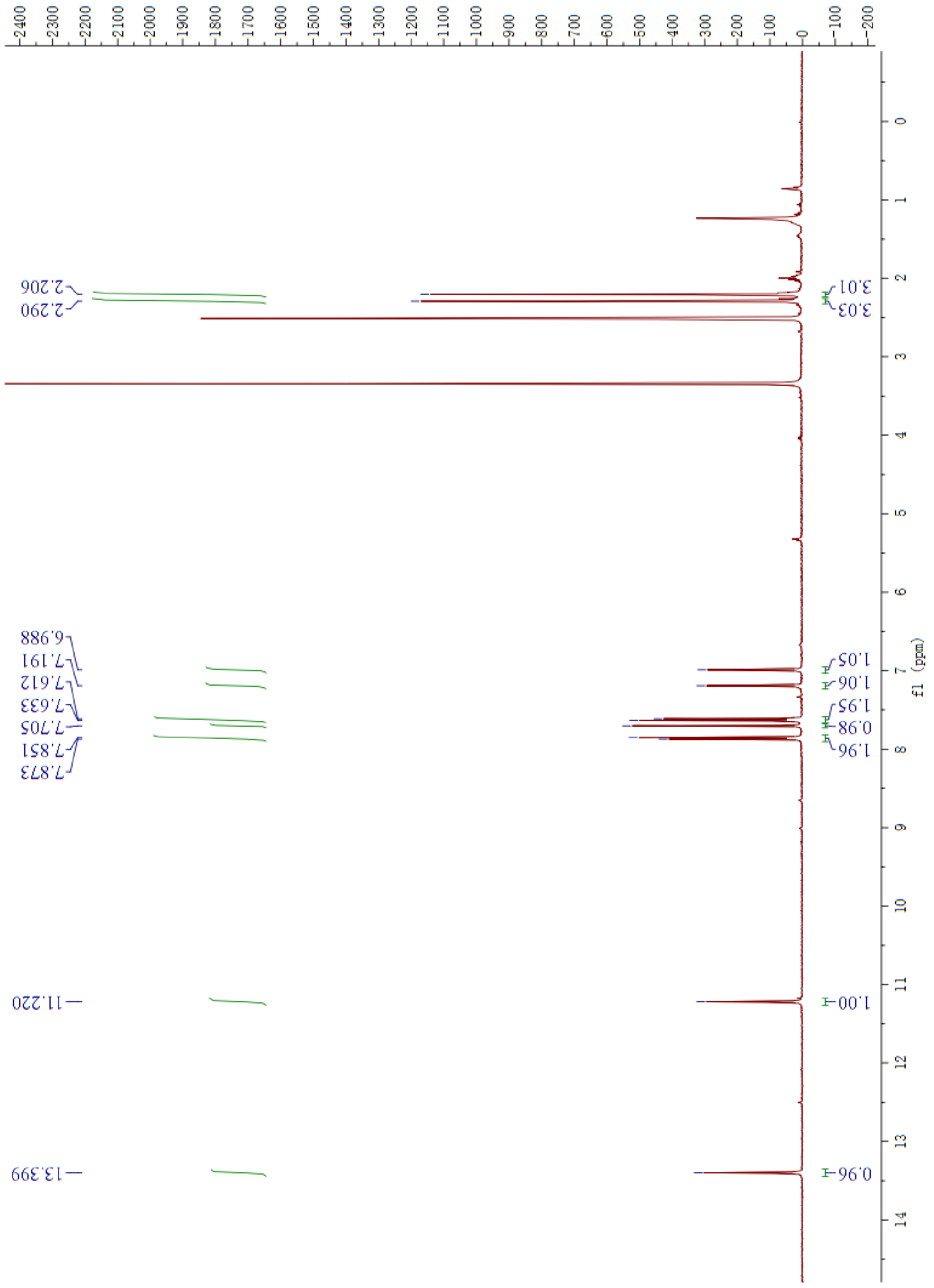

Figure S 15: ^{1}H NMR of Compound **6h**

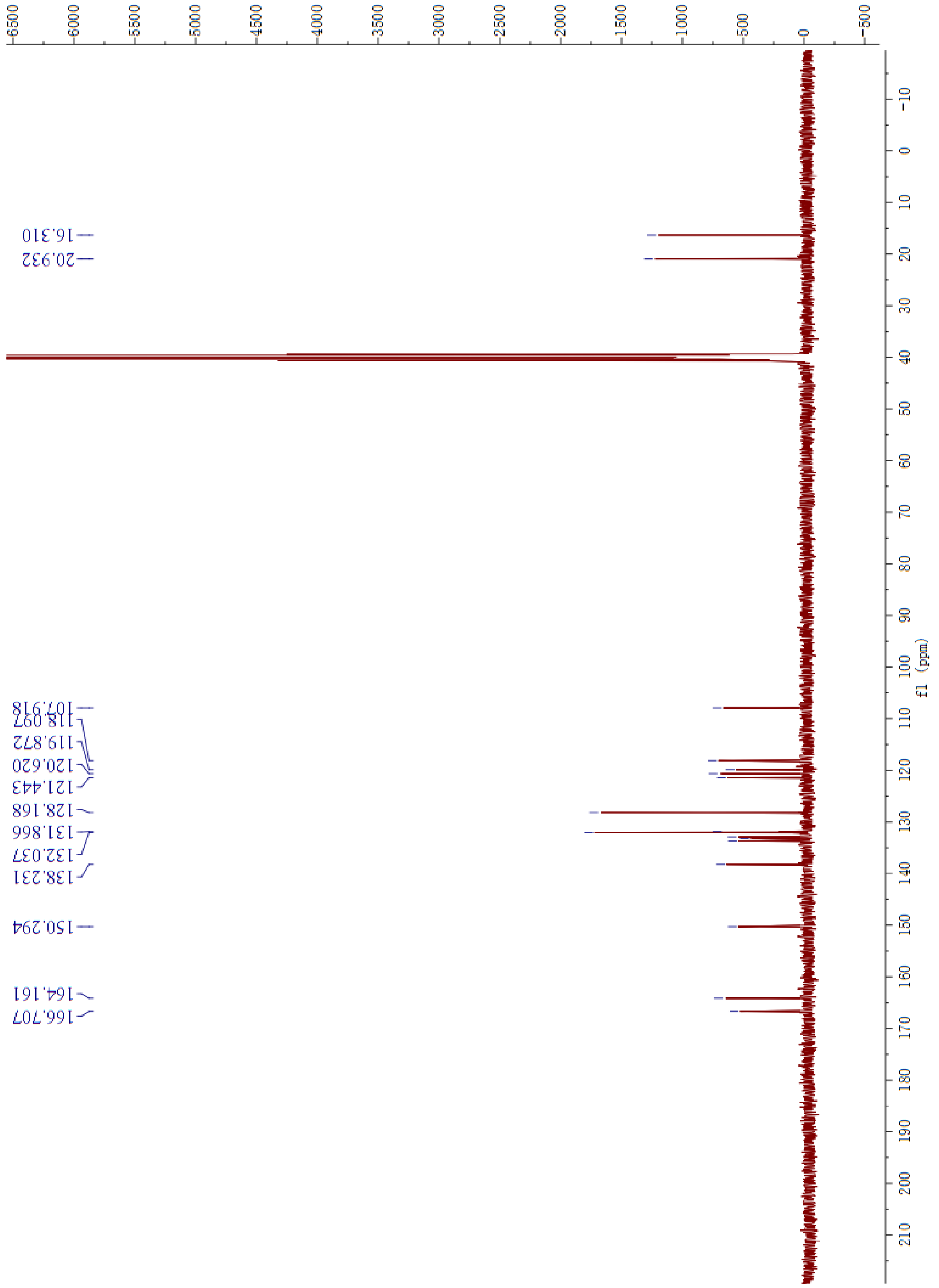

Figure S 16: ^{13}C NMR of Compound **6h**

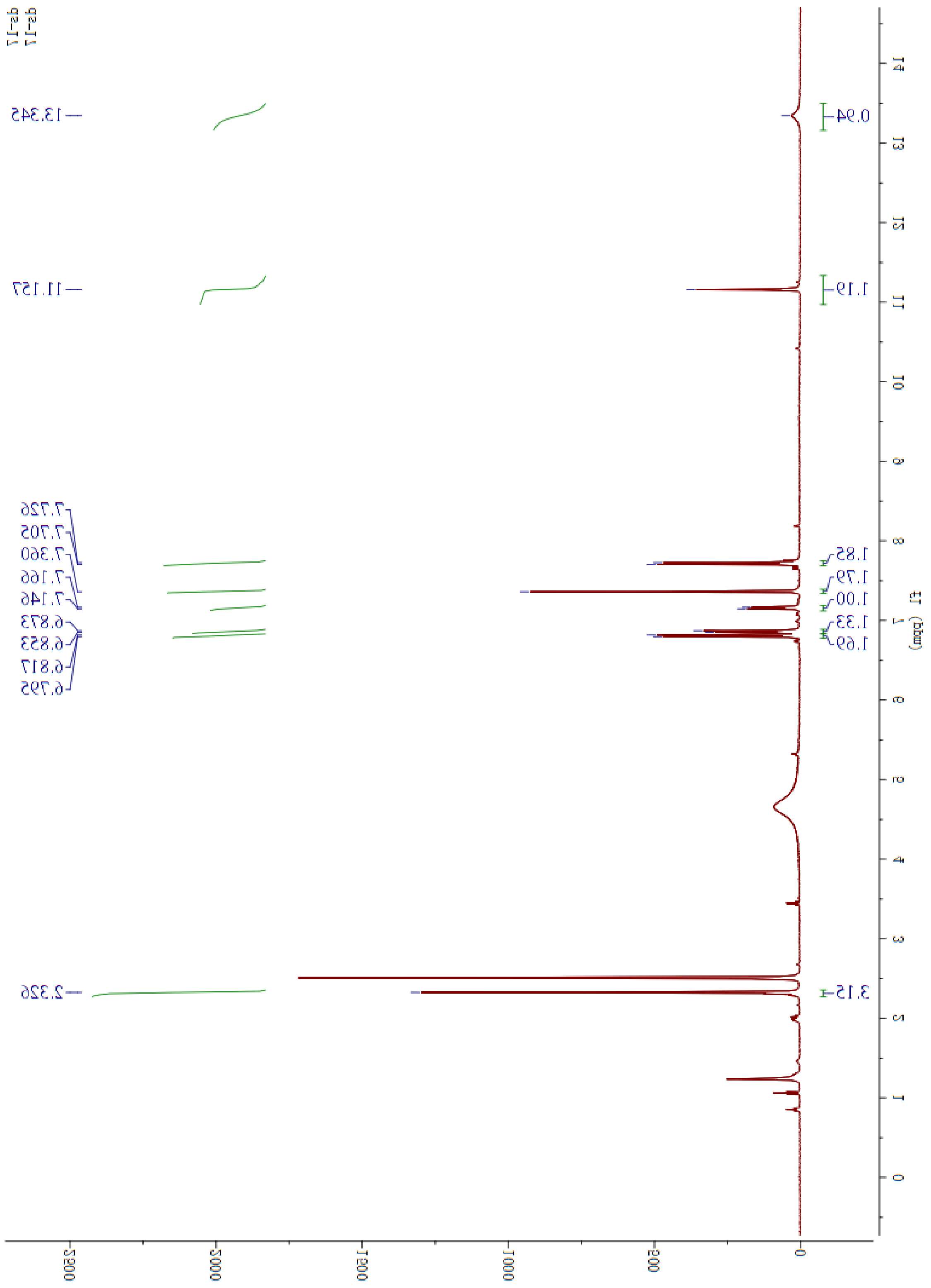

Figure S 17: ^{1}H NMR of Compound **6i**

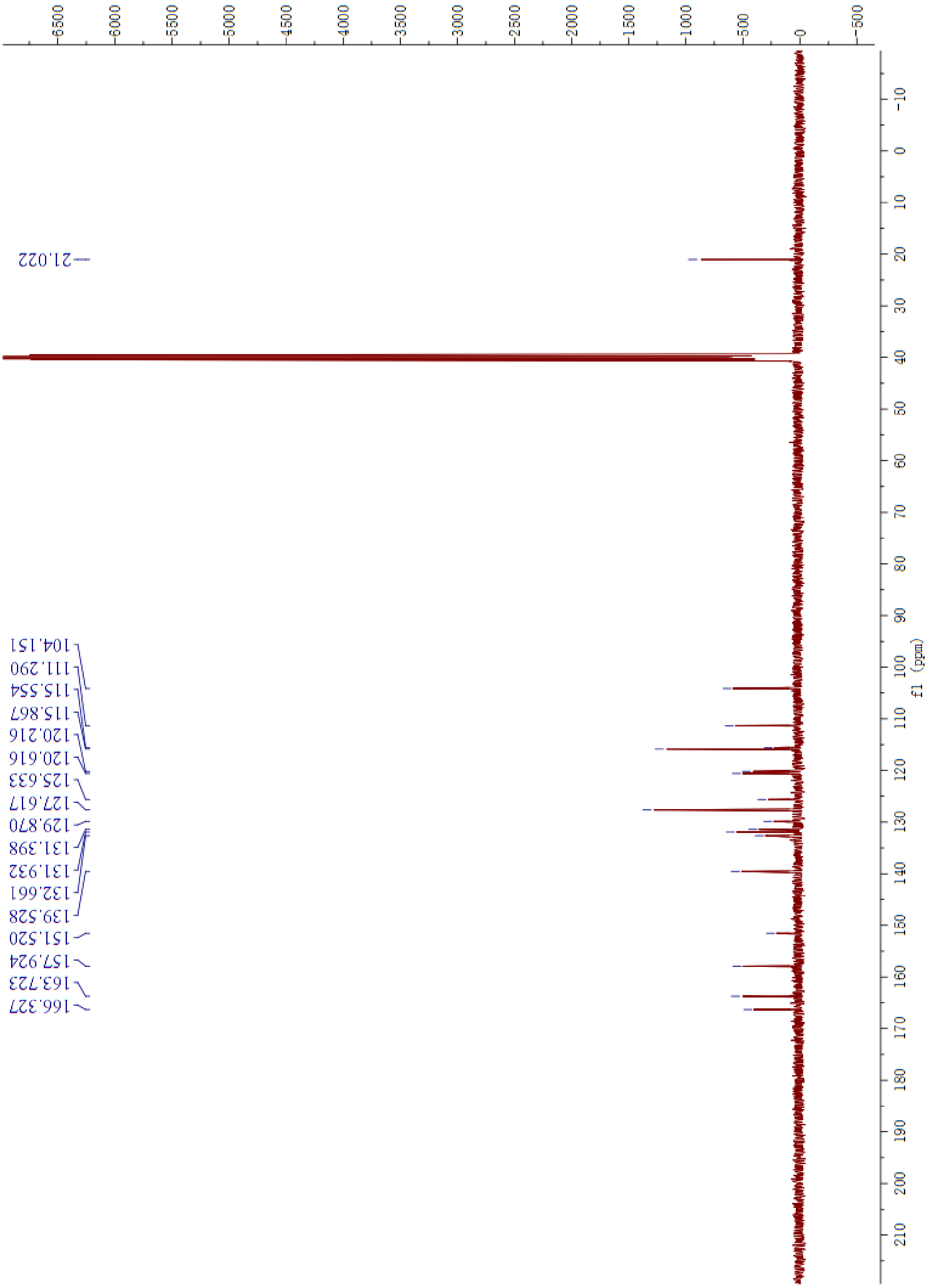

Figure S 18: ^{13}C NMR of Compound **6i**

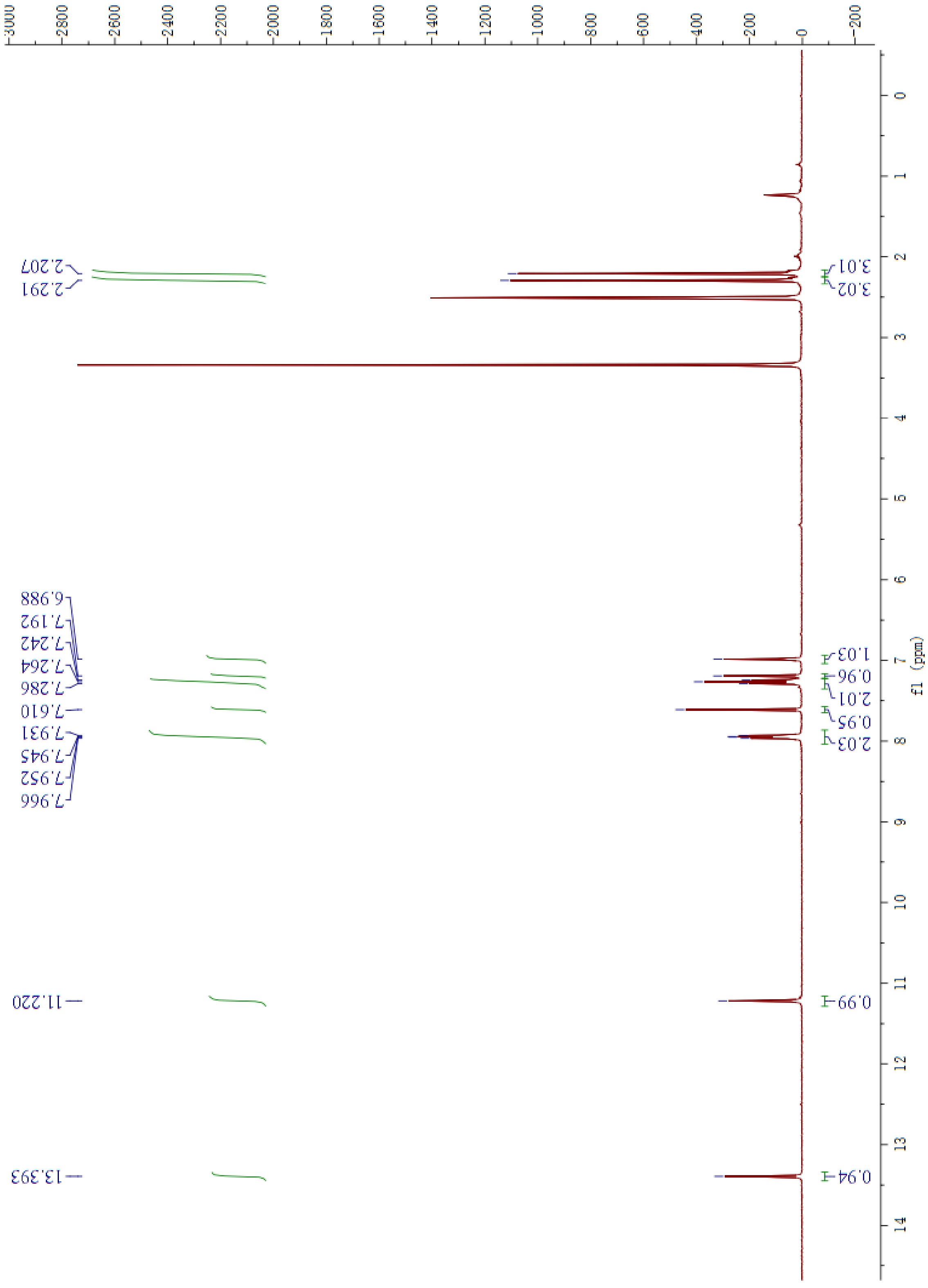

Figure S 19: ^{1}H NMR of Compound **6j**

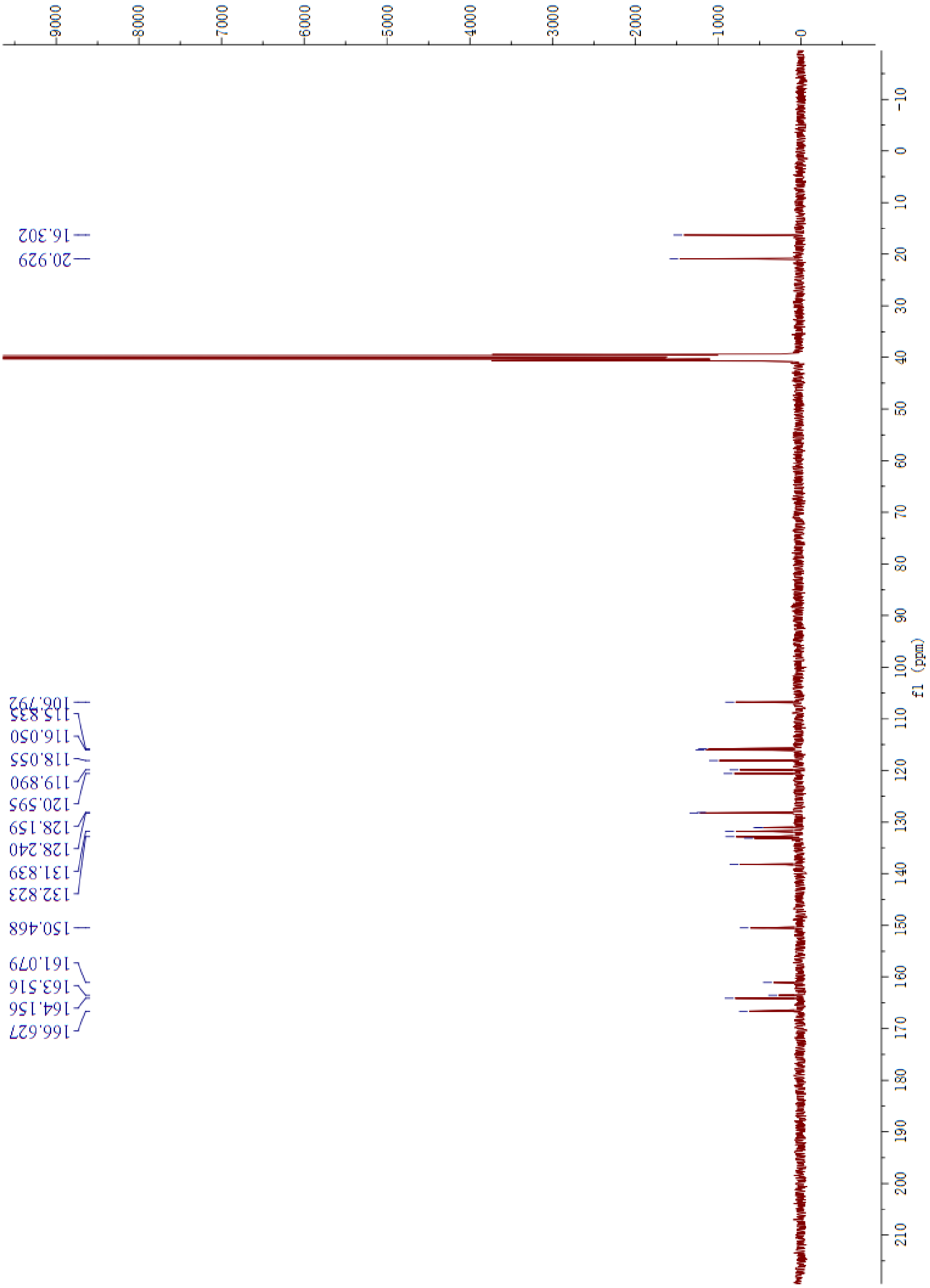

Figure S 20: ^{13}C NMR of Compound **6j**

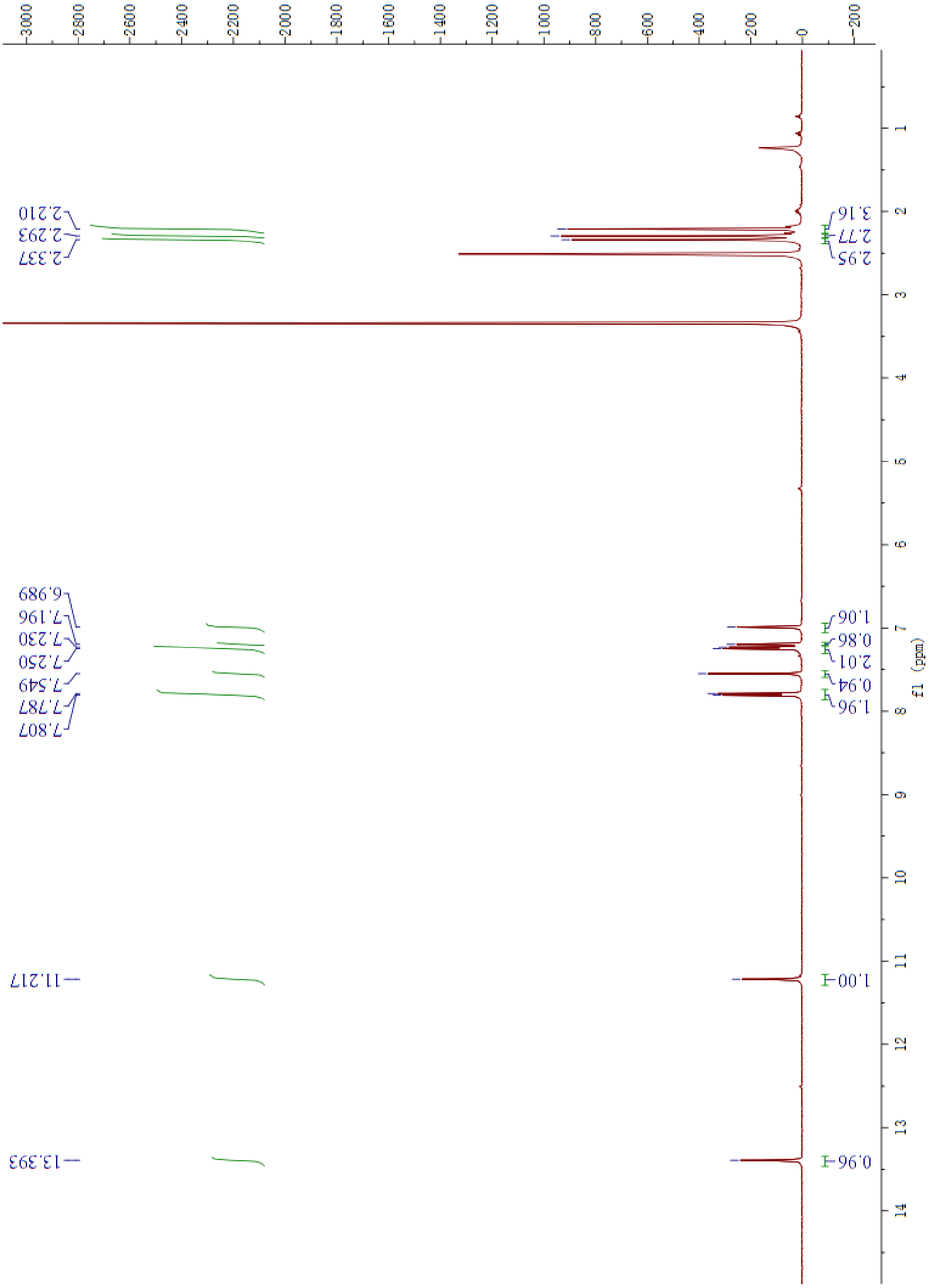

Figure S 21: ^{1}H NMR of Compound **6k**

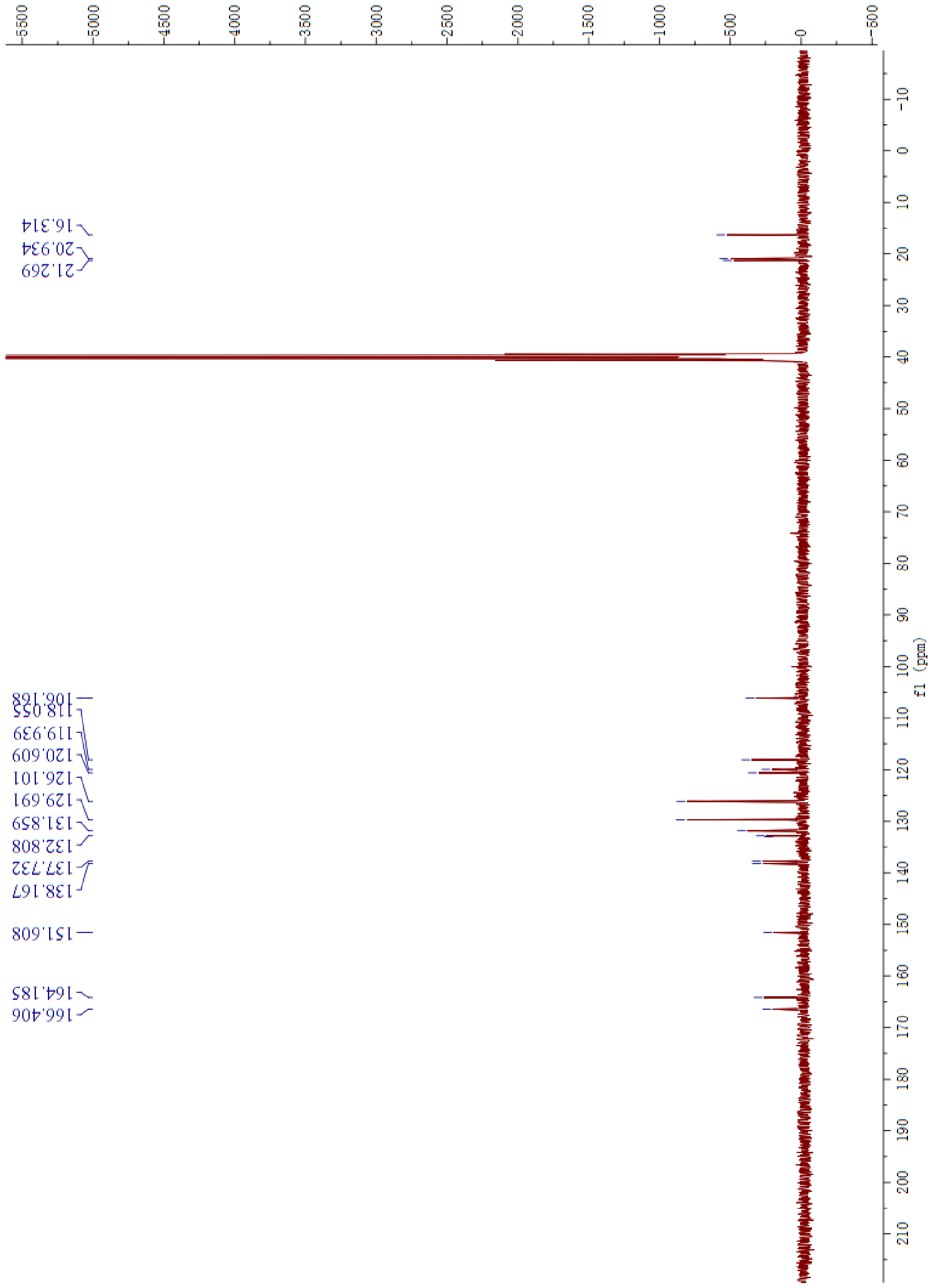

Figure S 22: ^{13}C NMR of Compound **6k**

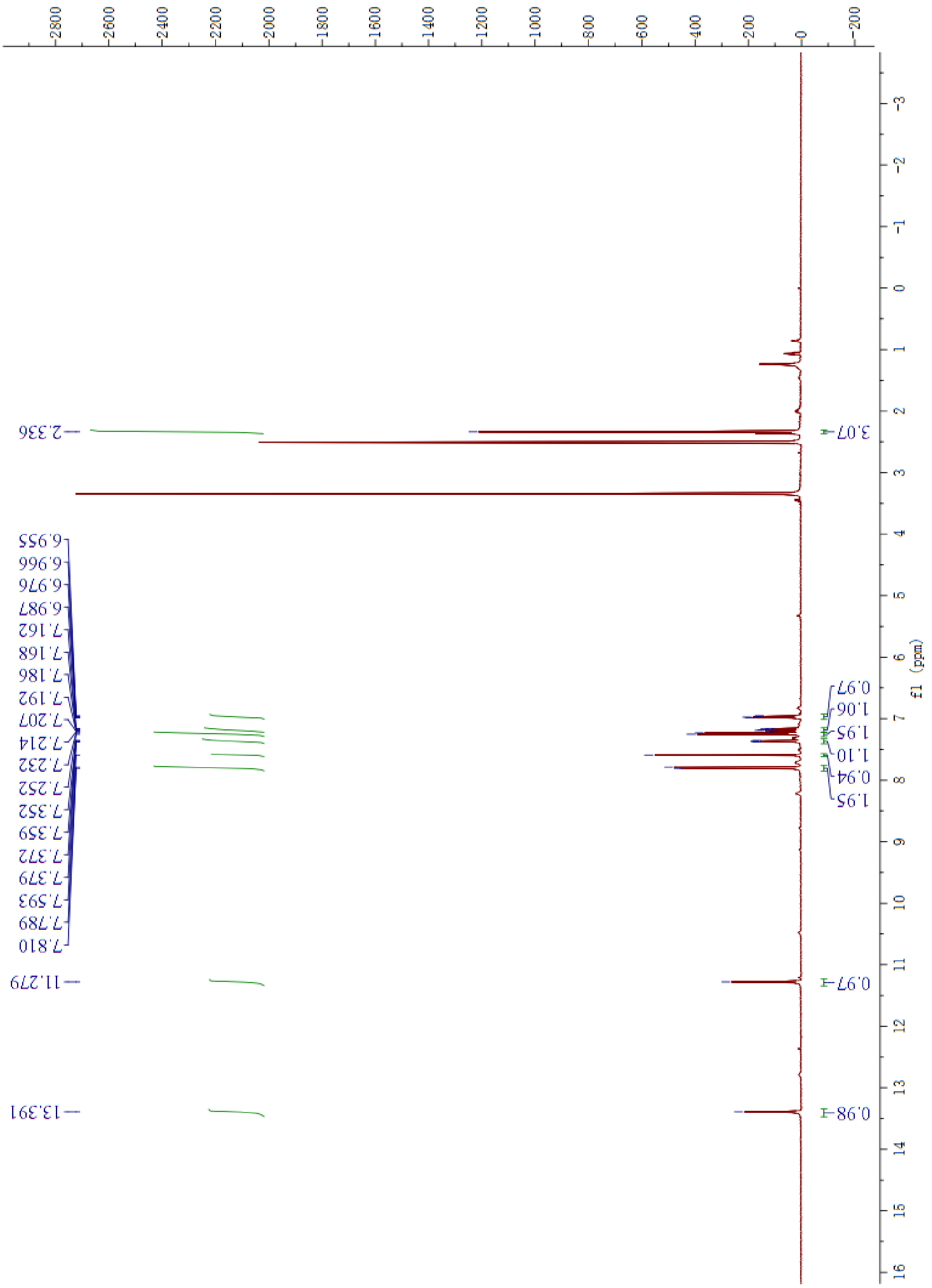

Figure S 23: ^{1}H NMR of Compound **6l**

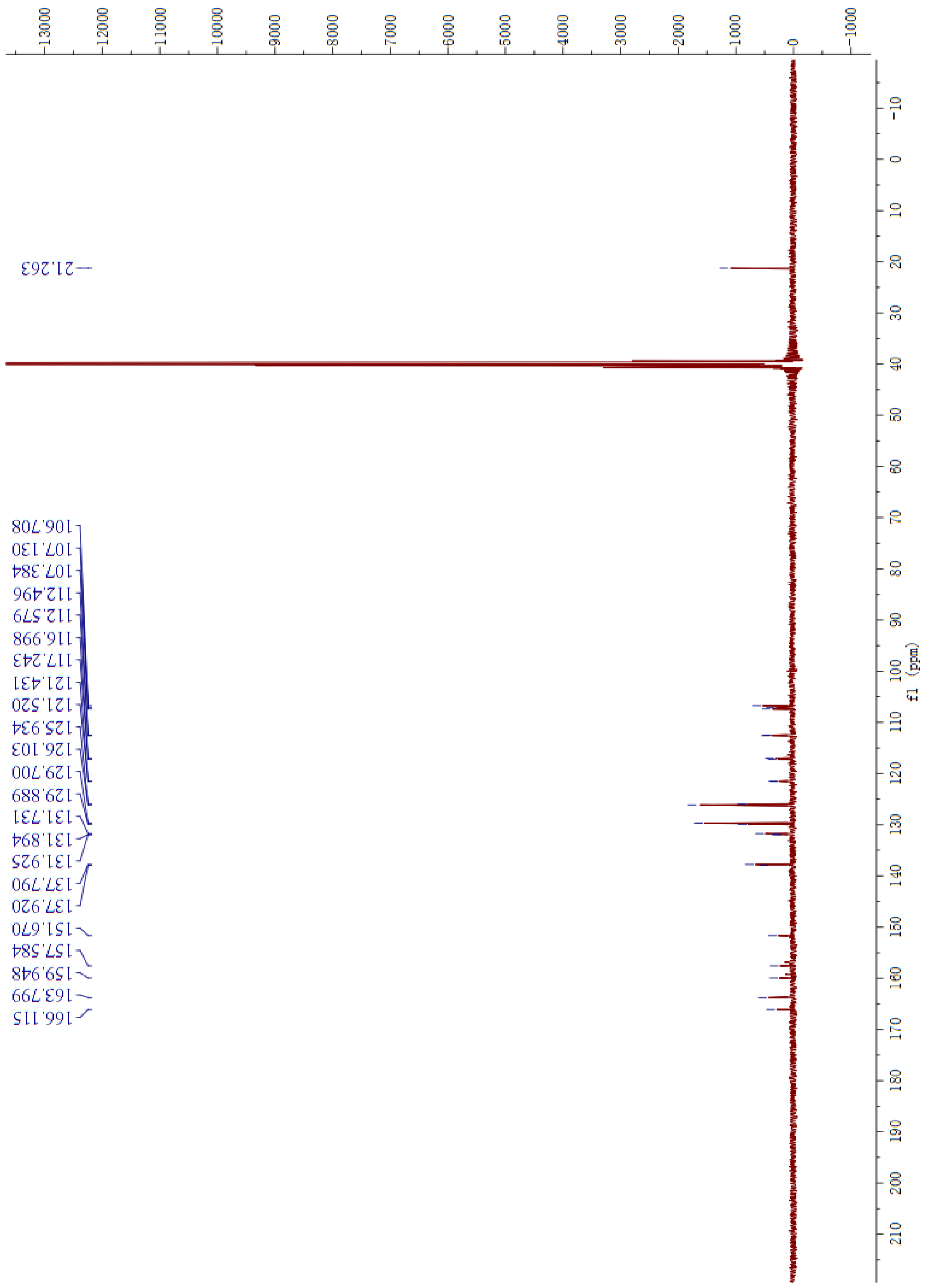

Figure S 24: ^{13}C NMR of Compound **6l**

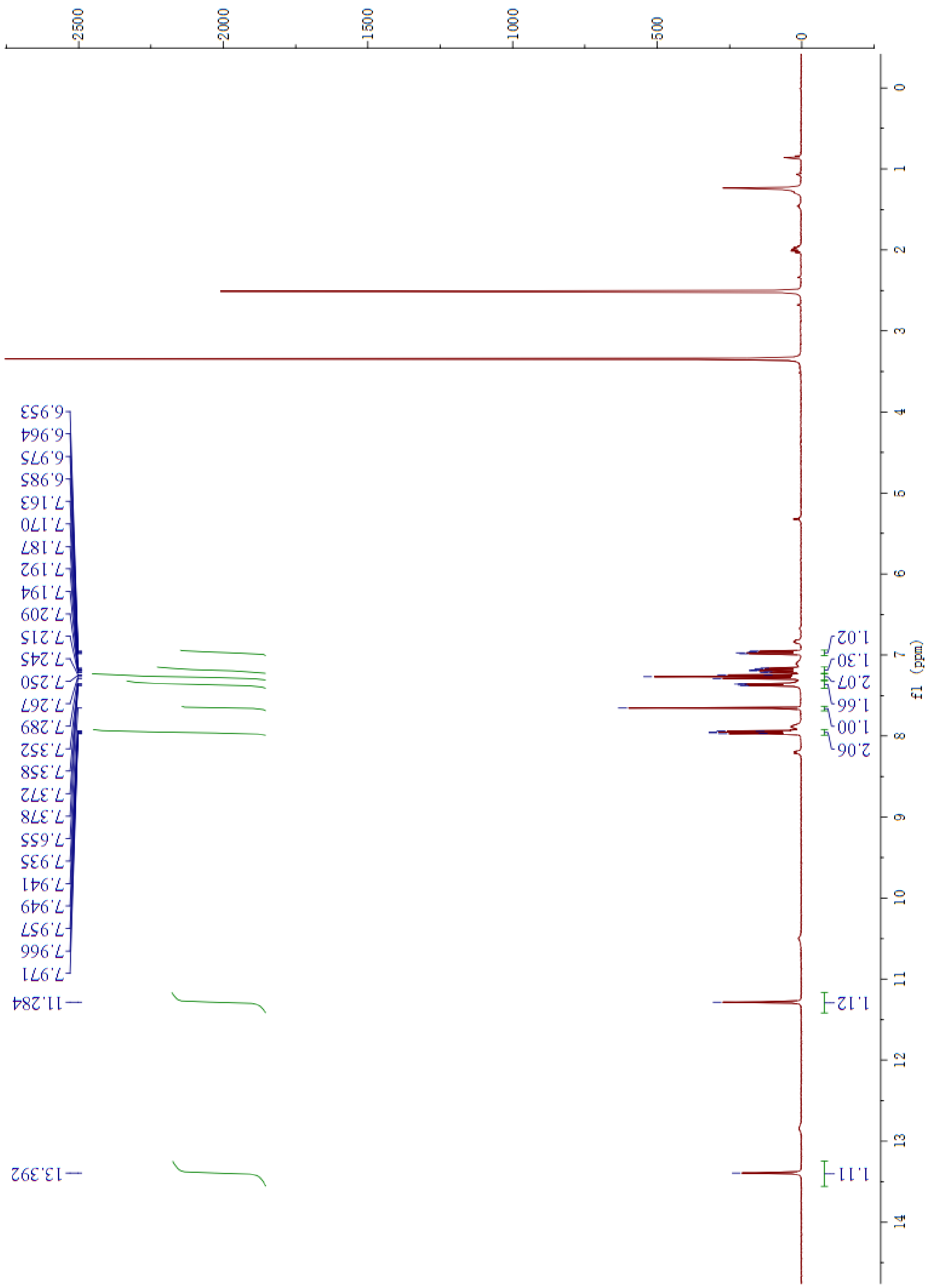

Figure S 25: ^{1}H NMR of Compound **6m**

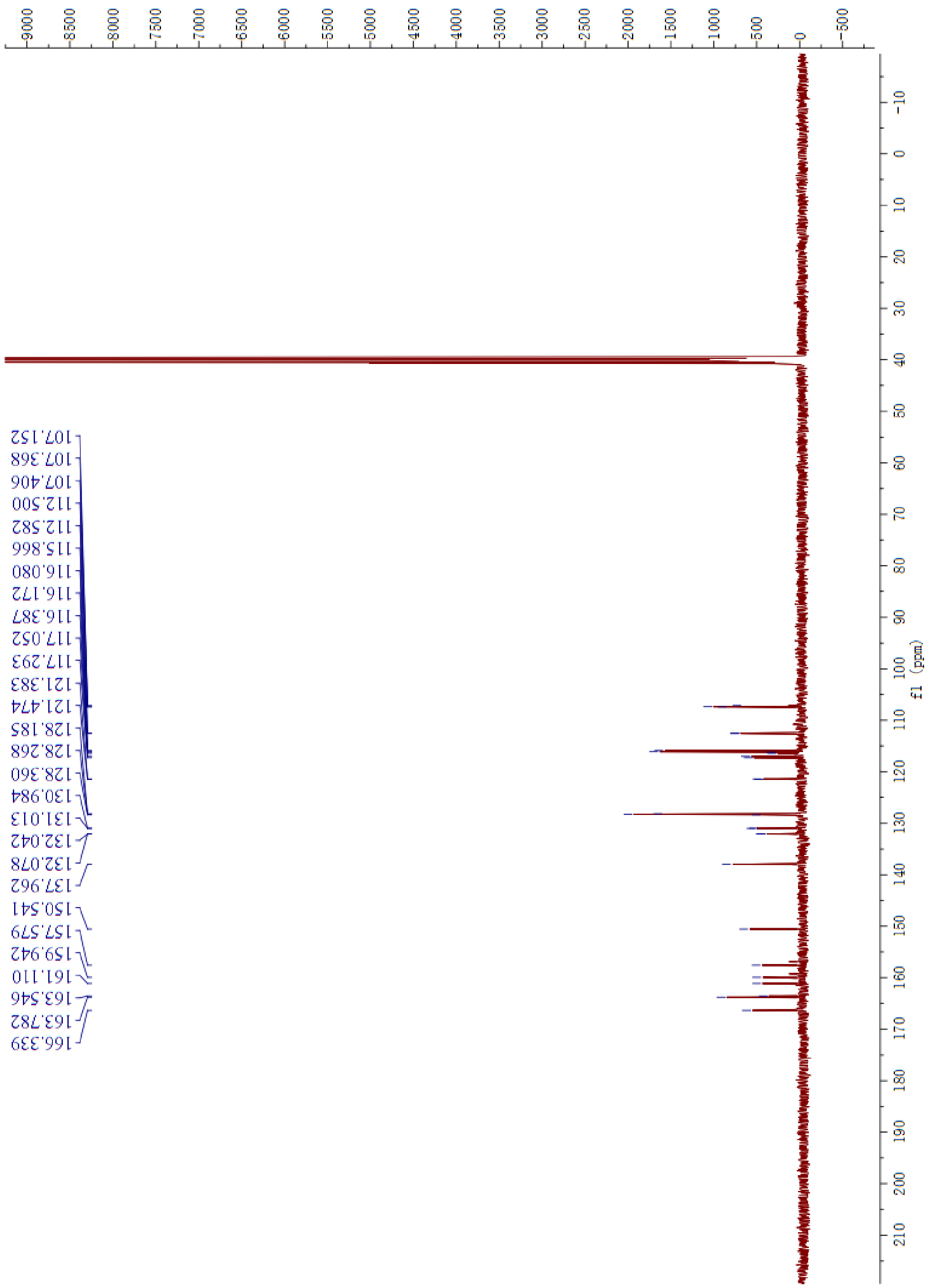

Figure S 26: ^{13}C NMR of Compound **6m**

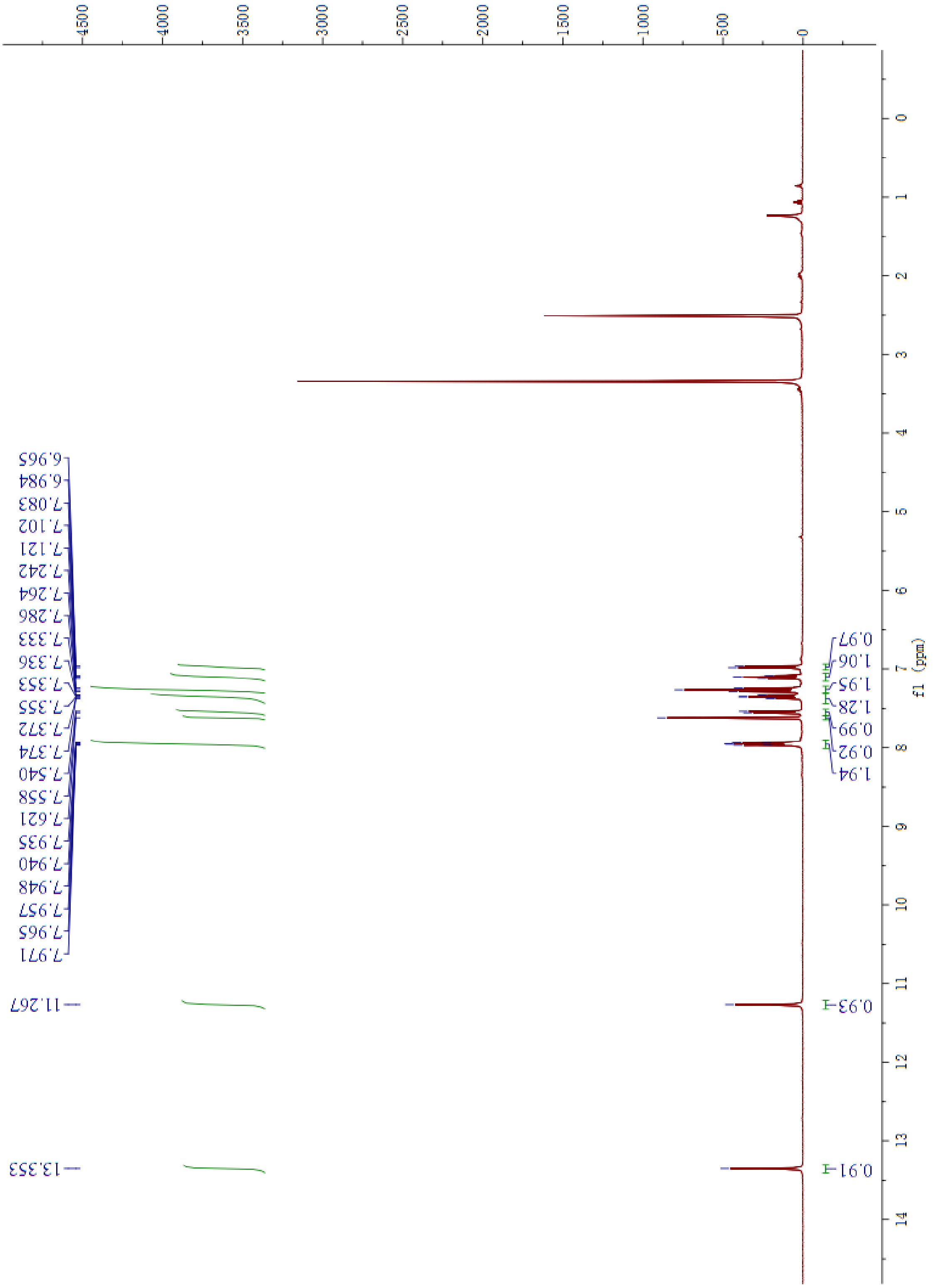

Figure S 27: ^{1}H NMR of Compound **6n**

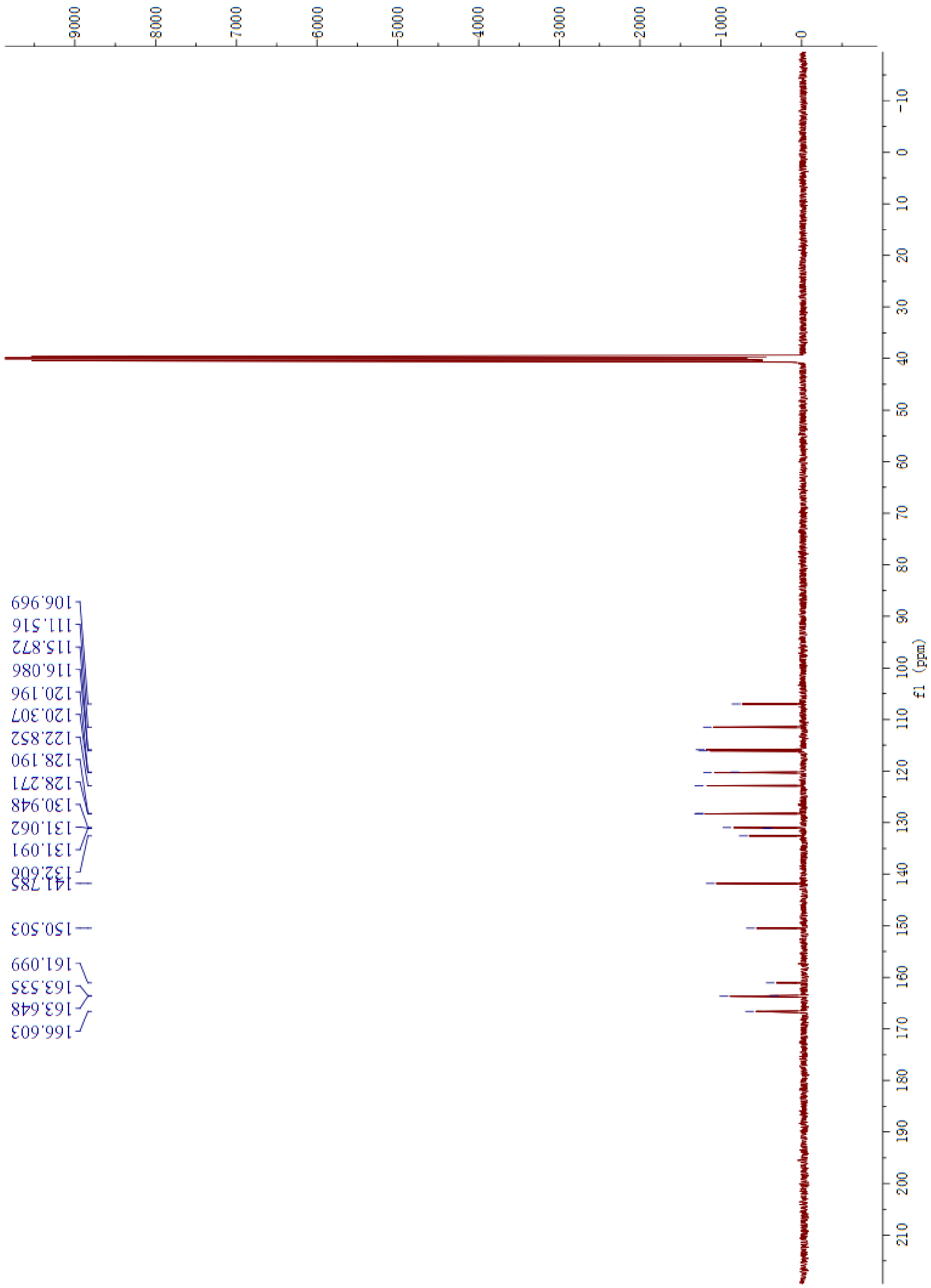

Figure S 28: ^{13}C NMR of Compound **6n**

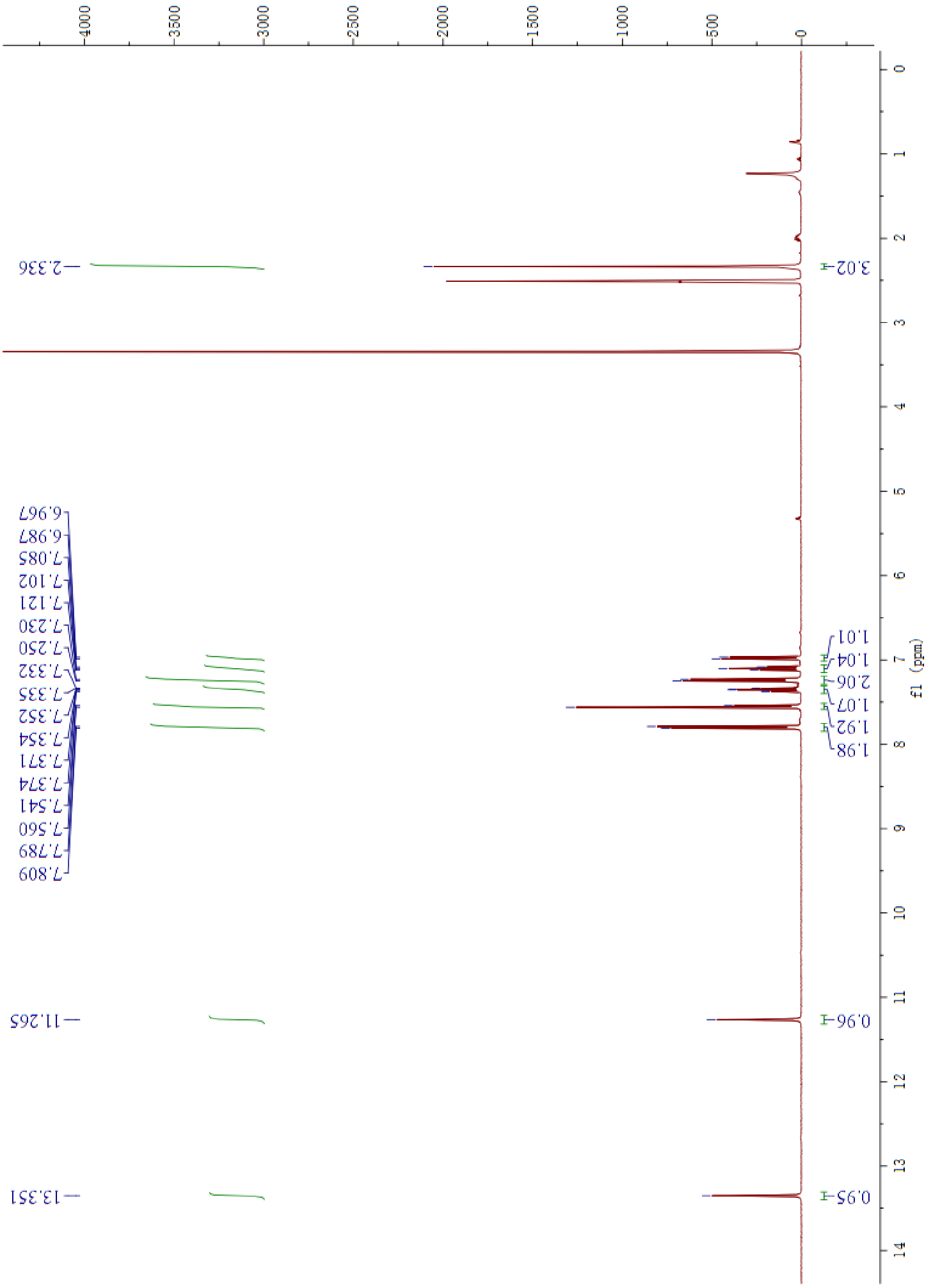

Figure S 29: ^{1}H NMR of Compound **6o**

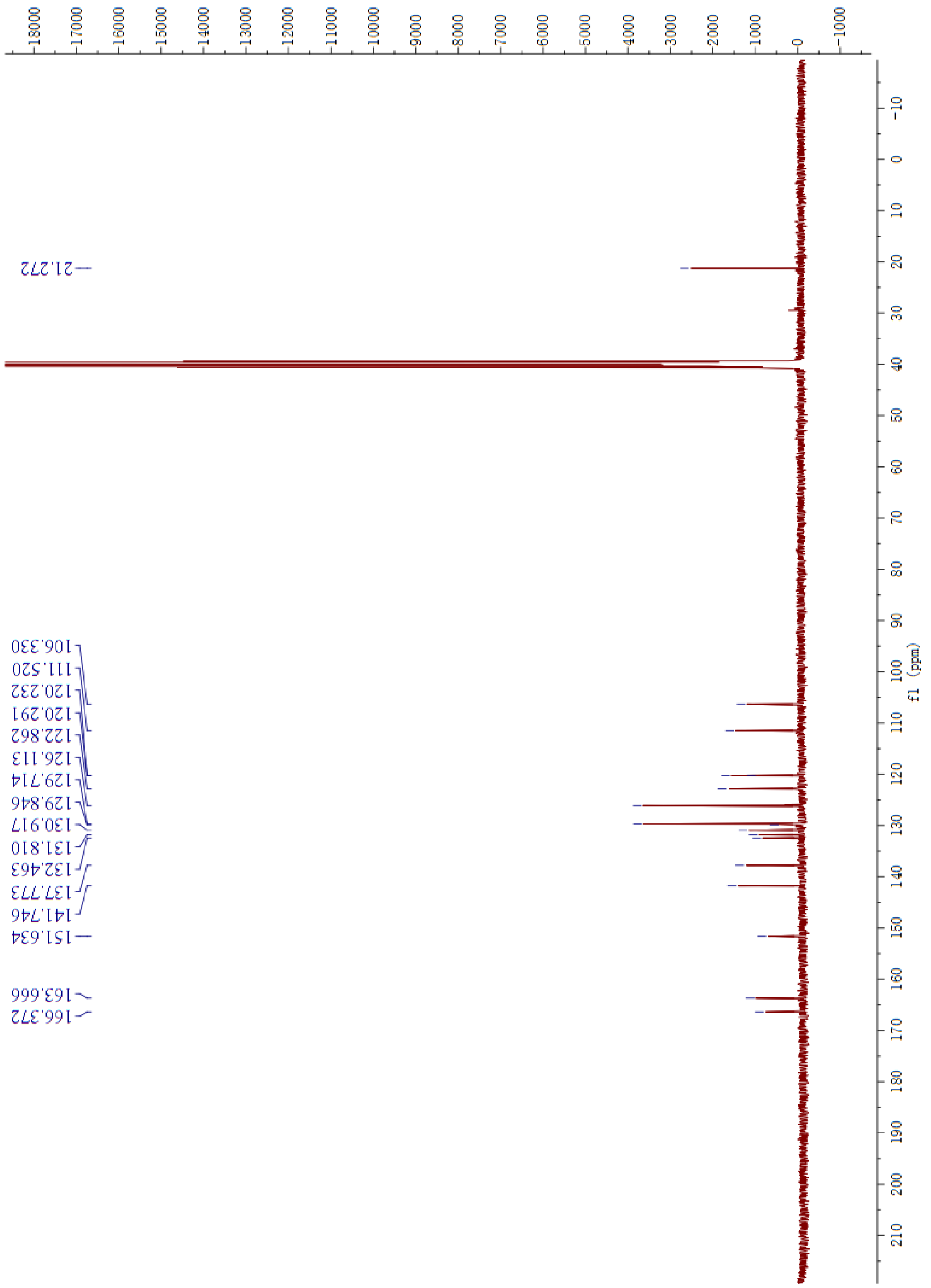

Figure S 30: ^{13}C NMR of Compound **6o**

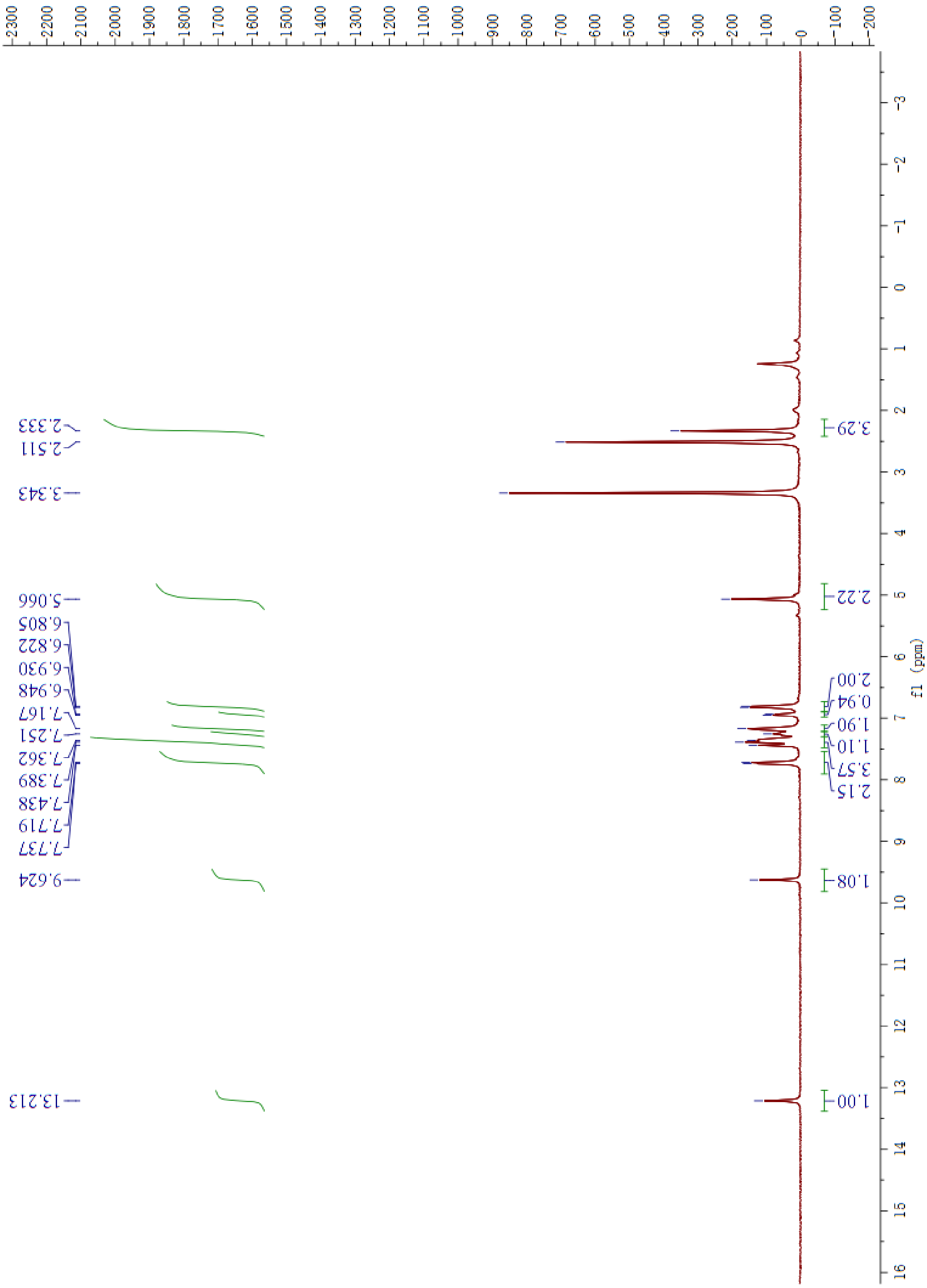

Figure S 31: ^{1}H NMR of Compound **6p**

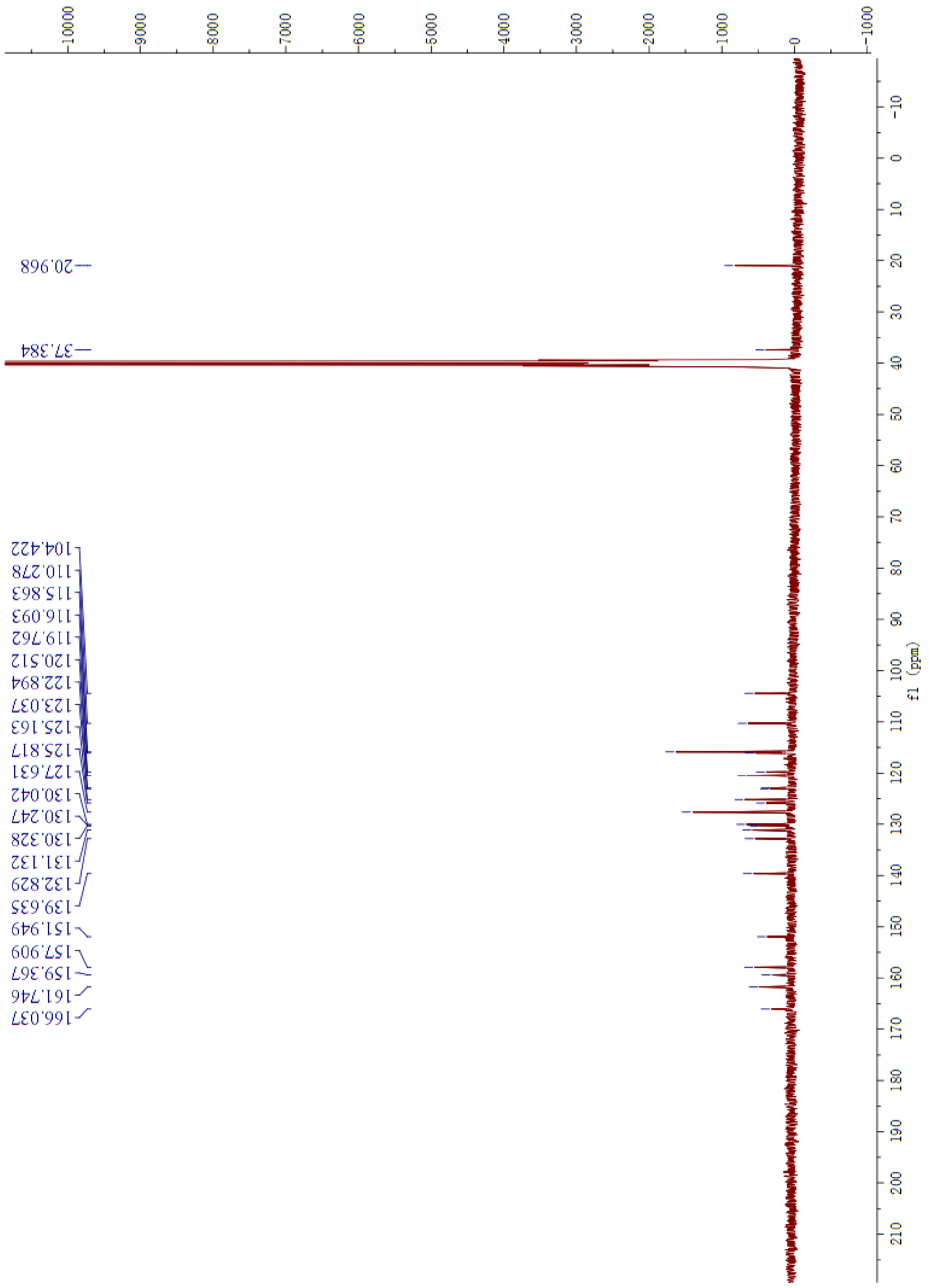

Figure S 32: ^{13}C NMR of Compound **6p**
